# The expression of Hexokinase 2 and its hub genes are correlated with the prognosis in glioma

**DOI:** 10.1186/s12885-022-10001-y

**Published:** 2022-08-18

**Authors:** Yishan Huang, Fan Ouyang, Fengxia Yang, Ning Zhang, Weijiang Zhao, Hongwu Xu, Xiaojun Yang

**Affiliations:** 1 Guangdong Provincial Key Laboratory of Infectious Disease and Molecular Immunopathology, Shantou University Medical College, Shantou, China; 2 Cell Biology Department, Wuxi School of Medicine, Jiangnan University, Wuxi, China; 3 Department of Neurosurgery, The First Affiliated Hospital of Shantou University Medical College, Shantou, China; 4 Department of Anthropotomy/Clinically Oriented Anatomy, Shantou University Medical College, Shantou, China

**Keywords:** HK2, Biomarker, Glioma, Hub genes, Prognosis, Immune infiltration

## Abstract

**Background:**

Hexokinase 2 (HK2) is an enzyme that catalyses the conversion of glucose to glucose-6-phosphate, which has been found to be associated with malignant tumour growth. However, the potential immunological and clinical significance of HK2, especially in terms of prognostic prediction for patients with glioma, has not been fully elucidated.

**Methods:**

To investigate the expression, immunological and clinical significance of HK2 in patients with glioma, several databases, including ONCOMINE, TIMER2.0, GEPIA, CGGA, UCSC, LinkedOmics, Metascape, STRING, GSCA, and TISIDB, as well as biochemical, cellular, and pathological analyses, were used in this study. In addition, we performed univariate, multivariate Cox regression and nomogram analyses of the hub genes positively and negatively correlated with HK2 to explore the potential regulatory mechanism in the initiation and development of glioma.

**Results:**

Our results demonstrated that HK2 was highly expressed in most malignant cancers. HK2 expression was significantly higher in lower grade glioma (LGG) and glioblastoma (GBM) than in adjacent normal tissue. In addition, HK2 expression was significantly correlated with clinical parameters, histological manifestations, and prognosis in glioma patients. Specifically, the data from The Cancer Genome Atlas downloaded from UCSC Xena database analysis showed that high expression of HK2 was strongly associated with poor prognosis in glioma patients. The LinkedOmics database indicated that HK2-related genes were mainly enriched in immune-related cells. In LGG and GBM tissues, HK2 expression is usually correlated with recognized immune checkpoints and the abundance of multiple immune infiltrates. Similarly, the Metascape database revealed that HK2-related genes were mainly enriched and annotated in immune-related pathways and immune cells. Further investigations also confirmed that the inhibition of HK2 expression remarkably suppressed metastasis and vasculogenic mimicry (VM) formation in glioma cells through regulating the gene expression of inflammatory and immune modulators.

**Conclusion:**

HK2 expression was closely associated with the malignant properties of glioma through activating multiple immune-related signalling pathways to regulate immune responses and the infiltration of immune cells. Thus, HK2 and its hub genes may be a potential target for the treatment of glioma.

**Supplementary Information:**

The online version contains supplementary material available at 10.1186/s12885-022-10001-y.

## Background

Gliomas, which are intrinsic brain tumours that originate from neuroglial progenitor cells [1], are the most malignant and aggressive form of brain tumours and account for the majority of brain cancer-related deaths [2]. According to the World Health Organization (WHO) criteria, gliomas can be mainly classified as low-grade gliomas (LGG; grades I - III) and high-grade diffuse gliomas (grade IV), which are also known as glioblastoma (GBM). Conventional therapies, including surgery, chemotherapy and radiotherapy, have achieved limited improvements in the prognosis of glioma patients [3, 4]. Previous studies indicated that immunotherapy can be a promising strategy for the treatment of malignant tumours, especially tumours derived from the central neural system, because of their penetrability to the blood-brain barrier [1, 5]. However, clinical trials have indicated that the effect of these strategies is still limited for the treatment of gliomas. Several recent reports revealed that immune checkpoints might be safely targeted with high antitumour efficacy in glioma and achieve better anticancer efficacy through dual targeting with a variety of inhibitory molecules [6, 7].

Hexokinases (HKs) catalyse the first committed step in glucose metabolism [8]. A recent study indicated that HKs are involved in metabolic flux through glycolysis in hyperglycaemia [9], while the abnormally regulated glucose metabolism frequently results in increased glycolysis intermediates or glycolysis overload and ultimately induces tumorigenesis [10]. Several reports have demonstrated that highly expressed HKs (mostly of the HK2 isoform) are positively correlated with tumorigenesis in many malignant tumours, including glioma, bladder cancer, oral squamous cell carcinoma, colorectal cancer, and breast cancer [11–15]. In contrast, deletion of the *HK2* gene can significantly inhibit the proliferation of cancer cells in animal models [16]. HK2 might be crucial for the Warburg effect, cell metabolism, cell survival, gluconic metabolic reprogramming, immune response, and inflammation [17, 18]. However, the correlation between HK2 and immune checkpoints has not been fully elucidated [18, 19].

In this study, we investigated the potential roles of HK2 expression in predicting prognosis on the basis of various databases and explored the correlation with clinicopathological features and the possible regulatory mechanism in glioma. Specifically, GSEA, the functional annotations and the signalling pathways of HK2 and HK2 related genes, and the correlation among HK2-related genes were analysed by the LinkedOmics database, Metascape, and protein-protein interaction (PPI) network, respectively. In addition, the screening of hub genes of HK2-related genes and the correlation between hub genes and the prognosis in glioma patients were analysed by cytoHubba plug-in and GEPIA databases, respectively. The top five hub genes of HK2-related genes were further confirmed by univariate and multivariate Cox regression and nomogram analysis. In addition, further biochemical, cellular, and pathological assays also indicated that HK2 expression was closely associated with tumour metastasis and vasculogenic mimicry (VM) formation in glioma cells through regulating the gene expression of inflammatory and immune modulators, as well as poor prognosis in glioma patients. Therefore, our findings suggested that HK2 might be a potential biomarker for investigating glioma development and predicting prognosis in glioma patients.

## Methods

### Analyses of differential expression of HK2 in various malignant tumours and glioma

To study the potential functions of HK2 in tumorigenesis, the ONCOMINE database (www.oncomine.org), which is an integrated online cancer microarray database for DNA/RNA sequence analyses [20], was first used to investigate the transcriptional expression of HK2 between different cancer tissues and their adjacent normal tissues. Differences in transcriptional expression were compared by Student’s t-test. The cut-off *P-*value and fold change were as follows: *P*-value is 0.01; fold change is 2; gene rank is 10%, and data type is mRNA.

To investigate the expression of HK2 in different glioma histologies, the choices were as follows: gene is HK2; analysis type is cancer vs. adjacent normal tissue; cancer type is glioblastoma; data type is mRNA; *P*-value < 0.05; gene rank is top 10%. The correlation between immune infiltrates and the HK2 expression level in glioma of the Cancer Genome Atlas (TCGA) cohort was analysed by tumour immune Estimation Resource 2.0 (TIMER2.0; http://timer.cistrome.org/) [21]. The correlation of HK2 expression between glioma and adjacent normal tissue was analysed by using the GEPIA database (http://gepia.cancer-pku.cn/) [22].

### Chinese glioma genome atlas (CGGA) and UCSC Xena databases

The correlation between clinicopathological parameters and the mRNA level of HK2 in glioma patients was analysed by using the CGGA database (http://www.cgga.org.cn/), which is a user-friendly platform for data storage and analysis to explore brain tumour datasets from over 2000 specimens from Chinese cohorts. This database includes whole-exome sequencing (*n* = 286), DNA methylation (*n* = 159), mRNA sequencing (*n* = 1018), mRNA microarray (*n* = 301), and matched clinicopathological information, including gender, age, histologic grade, histology, IHD status, 1p/19q co-deletion and progression status [23].

To study the potential roles of HK2 expression in glioma, we investigated the correlation between HK2 expression and clinicopathologic characteristics, as well as the various prognostic parameters in patients with glioma (*n* = 669), by using a genome-related UCSC Xena (http://xena.ucsc.edu/) database [24].

### Survival analysis

The correlation between the expression of HK2 (or hub genes) and four prognostic indices, including overall survival (OS), progression-free survival (PFS), disease-free survival (DSS), and disease-free survival (DFS), was specifically analysed in brain tumours. In this context, the time from randomization to death (for any reason), the period between the beginning of treatment and the observation of disease progression or death from any cause in patients with tumour diseases, the terminal event was disease recurrence or death after treatment, and the death caused by a specific disease is the end event, were respectively defined as OS, PFS, DFS, and DSS. If it is not caused by a specific disease, it will not be included in the outcome index. The Kaplan-Meier plotter survival curve of HK2 (or hub genes) in TCGA dataset was analysed by the “survival” and “survminer” packages in R language (or GEPIA database). The receiver operating characteristic (ROC) curve was analysed by the “survival ROC” package. In addition, an immune-related prognostic model was established by using univariate and multivariate Cox regression analyses. The results were displayed using the forestplot package. All analytical methods above and R packages were performed using R software version v4.0.3.

### LinkedOmics database analysis

The LinkedOmics database (http://www.linkedomics.org) [25], which contains multi-omics data and clinical data for 32 cancer types and a total of 11,158 patients from TCGA, was used to analyse the HK2-related genes and the signalling pathways in glioma. Briefly, the parameters were as follows: cancer cohort was glioma (LGG and GBM; *n* = 669); dataset was RNAseq; dataset attribute was HK2; RNAseq and statistical method was Pearson correlation method; selected tool was gene set enrichment analysis (GSEA); enrichment analysis was Kyoto Encyclopedia of Genes and Genomes (KEGG) pathway; rank criteria were from LinkFinder module. KEGG (Kyoto Encyclopedia of Genes and Genomes) (https://www.genome.jp/kegg/) is a knowledge base for systematic analysis of gene functions, and it is the most well-known and universal signal pathway database [26].

### Enrichment analysis and PPI network

Metascape (http://metascape.org), an integrated set of more than 40 gene function annotation databases [27], was used to investigate the enrichment analysis of HK2-related genes obtained from LinkedOmics (|Pearson’s rho| ≥ 0.5, *P* < 0.05). In addition, the analysis included gene ontology (GO) and KEGG enrichment analysis. We set min overlap as 3, min enrichment as 1.5, and *P* < 0.05 as significant.

To analyse the protein–protein interactions, the STRING online database (http://string-db.org; version: 11.0) was used to build a PPI network [28]. The molecular composite detection plug-in cytoHubba within Cytoscape was used to cluster the potential PPI network [29].

### Evaluation of the correlation between immune infiltration and HK2 expression in glioma

The correlation between HK2 expression and immune infiltration in glioma was analysed using the TIMER2.0 database, which is a comprehensive resource for systematically analysing immune infiltrates in many malignant cancers [21]. In addition, Pearson analysis was performed to assess the correlations between HK2 expression and immune checkpoints, as well as mismatch repair (MMR) proteins. In addition, TISIDB (http://cis.hku.hk.TISIDB/) was performed to analyse the potential roles of HK2 in the regulation of immunomodulatory factors in glioma [30]. GSCALite database (http://bioinfo.life.hust.edu.cn/GSCA/#/) is a cross type comprehensive cancer analysis database. It explores the gene set cancer analysis relate to mRNA expression, mutation, immune infiltration and drug resistance, including 33 types of cancer data of TCGA and GDSC. The database contains the analysis of genome (gene expression, Single Nucleotide Variation (SNV), Copy Number Variation (CNV) and DNA methylation) and immune genome (including 24 immune cells). The TCGA mRNA expression and DNA methylation data were obtained from UCSC Xena. DNA methylation profile was measured experimentally using the Illumina Infinium HumanMethylation450 platform (about 1% of all human methylation sites, and it covers most CpG islands and promoter regions). DNA methylation values, described as beta values, are recorded for each array probe in each sample via BeadStudio software. DNA methylation beta values are continuous variables between 0 and 1, representing the ratio of the intensity of the methylated bead type to the combined locus intensity. Thus, the higher beta values represent higher level of DNA methylation, i.e. hypermethylation and lower beta values represent lower level of DNA methylation, i.e. hypomethylation. Finally, the correlations between the DNA methylation of HK2 or hub genes and immune infiltrates, as well as the potential roles of hub genes in the immune cell subset, were analysed by using the GSCALite database [31].

### Cells and cell culture

The human glioma cell line T98 cells and the normal human brain astrocyte cell line HEB cells, were purchased from China Center for Type Culture Collection (Shanghai, China). All the cells were cultured in DMEM medium (Gibco, Thermo Fisher Scientific, Waltham, MA) supplemented with 10% FBS (Gibco) and streptomycin/penicillin antibiotic mixture (Invitrogen, Thermo Fisher Scientific) at 37 °C with 5% CO_2_. The primary cell culture was slightly modified from the previously reported protocol [32–34]. The cell lines T98 and HEB cells were authenticated by short tandem repeat (STR) profiling of 15 loci and the amelogenin sex determination (X or XY) method (Promega, Madison, WI) according to our previous described [35]. The authenticated primary cell line (GBM1) that derived from GBM surgical specimens, was obtained from Procell Company (Wuhan, China). The primary cell line was maintained in primary serum-free cultures grown on laminin [36]. Before each experiment, the GBM1 cells were cultured in DMEM medium, supplemented with 10% FBS, streptomycin/penicillin antibiotic mixture, and 2 mM L-glutamine (Invitrogen) at 37 °C with 5% CO_2_. For transfection, the siRNAs targeting HK2 (si-HK2) were synthesized by RiboBio Biotech (Guangzhou, China). All cells were transfected with si-HK2 by using Lipofectamine 3000 reagent (Invitrogen, Thermo Fisher Scientific) according to the manufacturer’s instruction.

### Quantitative real-time PCR (qRT-PCR)

Total RNA of different groups of cells was extracted by using a Trizol kit (Invitrogen). The RNA was reverse transcribed using the StarScript II First-strand cDNA Synthesis Mix (GenStar, Shanghai, China). The subsequent real-time quantitative polymerase chain reaction was configured according to 2 × RealStar Green Fast Mixture instructions (GenStar). The RT-qPCR was detected using the ABI QS5 Real Time PCR software (ABI, Thermo Fisher Scientific) and normalized to actin by using 2^-ΔΔCt^. All primers were listed in Supplementary Table S1.

### Western blot

The protein samples were extracted from different groups of cells by using cracking buffer solution RIPA (Solarbio, Beijing, China) at 4 °C for 15 min. The supernatants were centrifuged at 15,000 rpm, and the total protein was quantified by the BCA Kit (Solarbio). Thereafter, the protein samples were separated on 10% SDS-polyacrylamide gels and transferred to 0.45 μm PVDF membranes (Millipore, Billerica, MA). The blots were blocked with 5% Bovine Serum Albumin (BSA) in PBS buffer for 1 h, and then incubated with the appropriate primary antibodies (1:2000) at 4 °C overnight. After washing, the blots were incubated with the corresponding secondary antibodies (1:5000) at room temperature for 2 h. The signals were visualized by using a SuperSignal West Pico Substrate Kit (Pierce, Thermo Fisher Scientific), and analysed by using ImageJ software (National Institutes of Health, Bethesda, MD). The anti-Hexokinase 2 (22029-I-AP) and anti-STAT3 (10253–2-AP) antibodies were obtained from ProteinTech Group Inc. (Rosemont, IL), the anti-p-AKT (#4060) and anti-AKT (#4691) antibodies were purchased from Cell Signaling Technology (Danvers, MA), anti-p-STAT3 (BM4835) and anti-GAPDH (BM1623) antibodies, and all secondary antibodies were from Boster Biological Technology Ltd. (Wuhan, China).

### Wound healing assays

All the cells were seeded and scratched with a 10 μL pipette tip in 24-well plate. The sound width was photographed at 0 and 24 h under a phase-contrast microscope, respectively.

### Transwell assay (cell invasion assay)

An 8-μm pore size of Falcon™ cell culture inserts (BD Biosciences, Franklin Lakes, NJ), coated with Matrigel (1:20, BD Biosciences) in the upper chambers, was placed in a 24-well Corning transwell microplates (Corning Incorporated, NY). A total 5 × 10^4^ cells were seeded into with serum-free DMEM medium. The DMEM medium with 10% FBS were filled in the bottom chambers. After 16 h of incubation, the cells on the upper surface were carefully removed with a cotton swab, and the membranes were fixed with 4% paraformaldehyde and stained with 0.1% crystal violet for 20 min, respectively. The invasive cells were examined under a phase-contrast microscope.

### Tube formation assay

The cold Matrigel (BD Biosciences) was added into a μ-Slide angiogenesis (ibidi GmbH, Gräfelfing, Germany) with a pre-chilled micropipette, and incubated 5% CO2-humidified incubator at 37 °C for 1 h for solidification. The cells (5 × 10^4^) were seeded in solidified Matrigel and incubated with 2% FBS for 3 h. The length of tubes in each field was photographed and the average of tubules from 3 random fields of view.

### Tissue microarray (TMA) and immunohistochemistry (IHC)

The glioma TMA, which was obtained from Outdo Biotech Ltd. (Shanghai, China), was stained with anti-Hexokinase 2 polyclonal antibody (1:50; 22,029-I-AP, Proteintech Group Inc.) at 4 °C overnight and analysed with Image-Pro Plus software (Media Cybernetics, Rockville, MD).

### Statistical analysis

The overview of our workflow was shown in Supplementary Fig. S1. The clinical significance of HK2 expression in glioma patients was further validated in the ONCOMINE, GEPIA, and TIMER2.0 databases. Briefly, the correlation between HK2 and hub gene expression was analysed by the GEPIA database. The HK2-related functional annotation and signalling pathways were identified using LinkedOmics and Metascape, respectively. In addition, the survival rates were displayed with HRs, 95% Cis, and log-rank *P*-value. Univariate and multivariate Cox regression was used to analyse the correlation between the mRNA level of hug genes and the prognosis of glioma patients. Statistical analyses of group differences were performed by using Student’s *t* test and ANOVA. All experiments were conducted with three independent replicates and the results were presented as means ± SEM. The differences in patient’s prognosis between different groups were analysed with Kaplan-Meier analyses with log-rank tests. All data were analysed by Rv4.0.3. *P* < 0.05 was regarded as statistically significant. All experiments were repeated three times with similar results.

## Results

### The expression of HK2 positively correlates with the malignancy of glioma

**Fig. 1:**
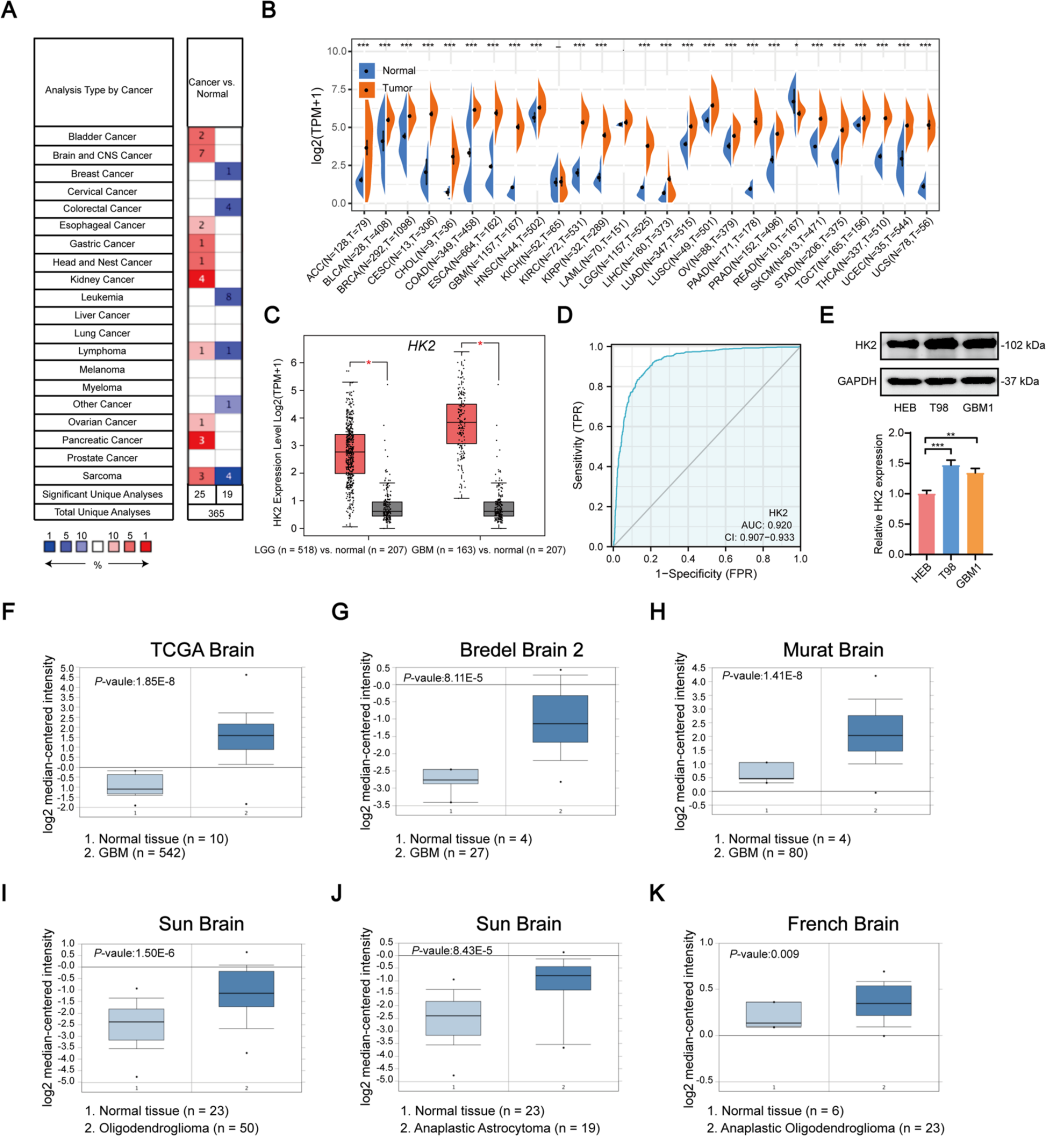
HK2 expression is positively correlated with the malignancy of human glioma. **A** The expression of HK2 in different cancers by using ONCOMINE database analysis. **B** The expression levels of HK2 in different cancer types from TCGA and GTEx datasets. **C** Compared with adjacent normal tissues, the expression of HK2 was much higher in LGG and GBM from GEPIA database. Box plots of differential expression of HK2 in different histologies of glioma. **D** The ROC curve analysis between patients and healthy controls to HK2. **E** The expression levels of HK2 expression in HEB, T98, and GBM1 cell lines. **F-K** The expression levels of HK2 were upregulated in different glioma subgroups (e.g., GBM, oligodendroglioma, anaplastic astrocytoma, and anaplastic oligoastrocytoma) from different datasets (e.g., TCGA, Bredel, Mural, Sun, and French datasets). Data shown as mean ± SD. *P < 0.05, **P < 0.01, ***P < 0.001

To get insight the potential roles of HK2 expression in tumorigenesis and tumour development, the ONCOMINE database was used to validate the expression level of HK2 in various malignant cancers. Compared with adjacent normal tissues, HK2 was found to be highly expressed in bladder cancer, oesophageal cancer, gastric cancer, head and nest cancer, kidney cancer, lymphoma, ovarian cancer, pancreatic cancer, sarcoma, and brain and central nervous system (CNS)-related cancer (Fig. 1A), suggesting that HK2 may play an important role in tumorigenesis. Moreover, the alignment between adjacent normal tissues from GTEx and tumour specimens from TCGA database reflected that high expression of HK2 was determined in most malignant cancers (Fig. 1B). Specifically, HK2 expression was significantly increased in ACC (adrenocortical carcinoma), BLCA (bladder urothelial carcinoma), BRCA (breast invasive carcinoma), CESC (cervical squamous cell carcinoma and endocervical adenocarcinoma), CHOL (cholangiocarcinoma), COAD (colon adenocarcinoma), ESCA (oesophageal carcinoma), GBM, HNSC (head and neck squamous cell carcinoma), KIRC (kidney renal clear cell carcinoma), KIRP (kidney renal papillary cell carcinoma), LGG, LIHC (liver hepatocellular carcinoma), LUAD (lung adenocarcinoma), LUSC (lung squamous cell carcinoma), OV (ovarian serous cystadenocarcinoma), PAAD (pancreatic adenocarcinoma), PRAD (prostate adenocarcinoma), SKCM (skin cutaneous melanoma), STAD (stomach adenocarcinoma), TGCT (testicular germ cell tumours), THCA (thyroid carcinoma), UCEC (uterine corpus endometrial carcinoma) and UCS (uterine carcinosarcoma). Notably, compared with the adjacent normal tissue, further GEPIA analysis then confirmed that HK2 expression was significantly increased in LGG and GBM tissues and positively correlated with the malignancy of gliomas (Fig. 1C). ROC showed that the expression of *HK2* mRNA in glioma and normal tissue was 0.92 (95% CI: 0.907–0.933) (Fig. 1D) and the best cut-off value of HK2 was 1.483 (TPM; Transcript per million). Similarly, our results also indicated significant upregulation of HK2 expression in T98 and GBM1 cells, compared with that in HEB normal control (Fig. 1E). In addition, the expression levels of HK2 were significantly upregulated in different glioma subgroups, including glioblastoma, oligodendroglioma, anaplastic astrocytoma, anaplastic oligoastrocytoma and anaplastic oligodendroglioma, in TCGA, Bredel, Mural, Sun, and French datasets (Fig. 1F-K, Supplementary Table S2), suggesting that HK2 may play an important role in glioma tumorigenesis and development.

### High expression of HK2 is associated with poor prognosis in glioma patients

We then evaluated the expression of HK2 with the CGGA database and several clinical parameters in glioma patients [37, 38]. Our results indicated that HK2 expression was positively correlated with histological manifestations (*P* = 5.9e-23), malignancy (*P* = 7.6e-19), IDH mutation (*P* = 2.6e-14), 1p/19q co-deletion (*P* = 2e-32), IDH mutation & 1p/19q co-deletion (*P* = 1.7e-26), and 1p/19q co-deletion in different grades (*P* = 0.012, *P* = 1.5e-18, and *P* = 6.6e-07, respectively) in glioma patients (Supplementary Fig. S2A-F). In addition, there was no significant difference between HK2 expression and gender (*P* = 0.28), age (*P* = 0.88), or progression (*P* = 0.084) in glioma (Supplementary Fig. S2G-I). Similarly, 669 LGG and GBM specimens in TCGA dataset from the UCSC Xena database (Supplementary Table S3) revealed that HK2 expression was closely correlated with the clinicopathological features of glioma patients, including malignancy (*P* < 0.001), IDH mutation (*P* < 0.001), O-6-methylguanine DNA methyltransferase (MGMT) promoter status (*P* < 0.001), transcriptome subtype (*P* < 0.001), histology (*P* < 0.001), and 1p/19q co-deletion (*P* < 0.001) (Supplementary Table S4).

**Fig. 2:**
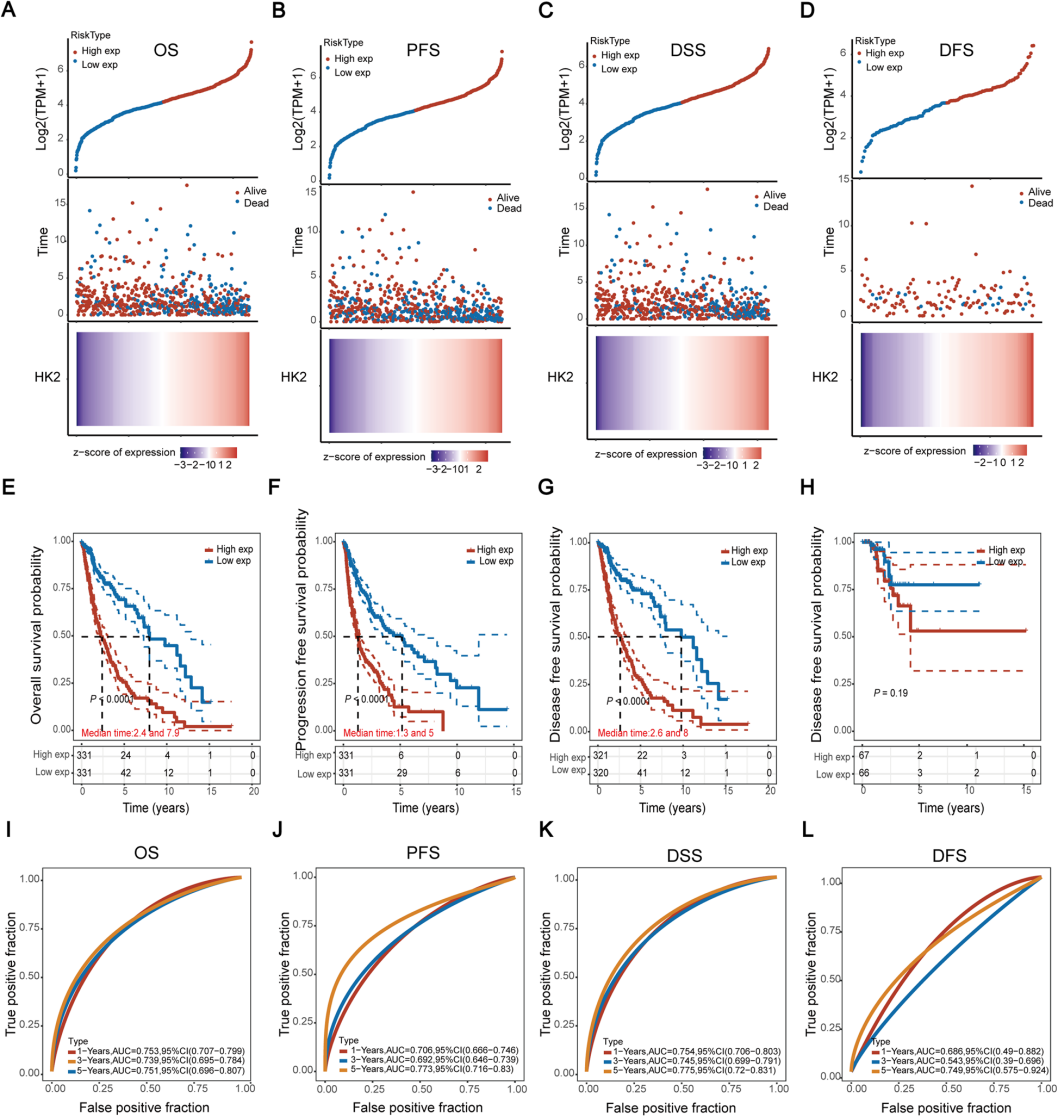
High expression of HK2 is associated with poor prognosis in gliomas. **A**-**D** Heatmap of HK2 expression profiles, risk type distribution, and survival time in glioma patients. **E**-**H** The correlation between HK2 expression and patient prognosis (OS, PFS, DSS and DFS) of glioma in TCGA database. **I**-**L** ROC curves for the 1-, 3-, and 5-year survival according to the HK2 risk signature in glioma. ****P < 0.0001

We further investigated the potential role of HK2 expression in predicting prognosis in glioma patients. Our results indicated that the survival rates decreased with increasing HK2 expression in OS, PFS, and DSS (Fig. 2A-C), whereas there was no correlation between HK2 expression and DFS in patients with gliomas (Fig. 2D). In addition, the mRNA level of HK2 was always accompanied by poorer OS (*P* < 0.0001), PFS (*P* < 0.0001), and DSS (*P* < 0.0001) (Fig. 2E-G) but not DFS (*P* = 0.19) in glioma patients (Fig. 2H). Analysis of TCGA dataset (*n* = 699) also illustrated the potential role of HK2 expression in predicting prognosis in glioma patients. As shown in Supplementary Table S5, compared with lower expression of HK2, high expression of HK2 was significantly associated with poor prognosis in glioma patients (*P* < 0.001). Multivariate Cox regression analysis indicated that histological grade (*P* < 0.001) and IDH mutation (*P* < 0.001) can be biomarkers for predicting poor survival when HK2 expression (*P* = 0.177), MGMT promoter status (*P* = 0.919), transcriptome subtype (*P* = 0.435), and 1p/19q co-deletion (*P* = 0.637) were considered. Furthermore, ROC curves of four prognostic models, including OS, PFS, DSS, and DFS, revealed that high expression of HK2 was positively correlated with poor prognosis in glioma (Fig. 2I-L).

**Fig. 3:**
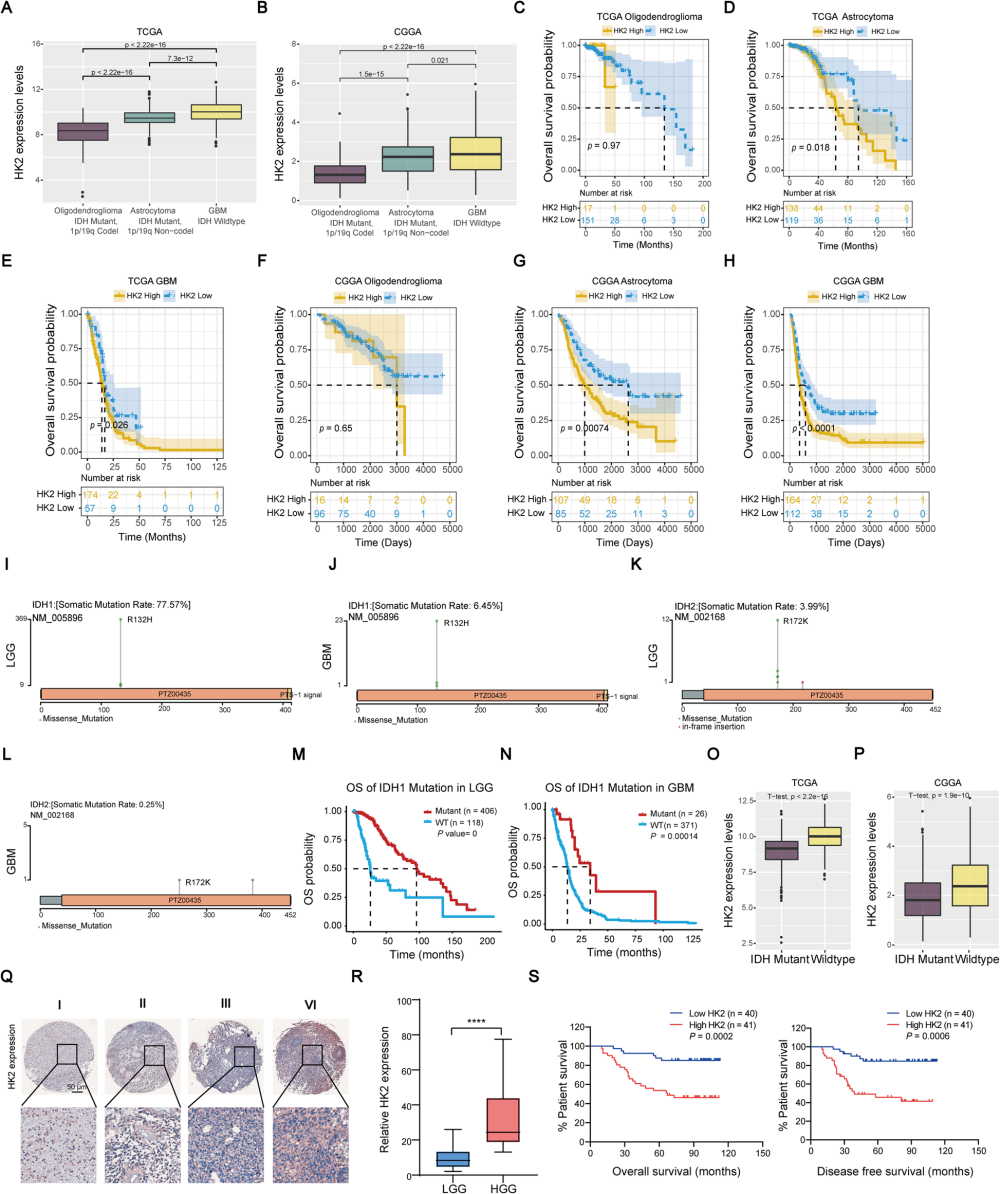
Evaluate HK2 gene expression with box plots according to clinical parameters in glioma patients using data from TCGA and CGGA databases. **A**-**B** The mRNA expression of HK2 was remarkably correlated with different histological manifestations of glioma in TCGA (**A**) and CGGA databases (**B**). **C**-**H** The higher mRNA expression of HK2 was associated with shorter OS in different histological manifestations of glioma. **I**-**J** Mutation rate of IDH1 in LGG (**I**) and GBM (**J**). **K**-**L** Mutation rate of IDH2 in LGG (**K**) and GBM (**L**). **M**-**N** IDH1 mutation in LGG (**M**) and GBM (**N)** was associated with OS. **O**-**P** HK2 expression was markedly decreased in the IDH mutation group compared to IDH wide type group in TCGA (**O**) and CGGA databases (**P**). **Q** Representative images of HK2 expression in glioma patient specimens at low (10 ×; upper panels) and high (100 ×; lower panels) magnification. **R** Quantification of the correlation between HK2 expression and malignancy of the glioma specimens. LGG, I and II grades; HGG, III and IV grades. **S** Overall and disease-free survival rates in low- (*n* = 40) or high-expression (*n* = 41) of HK2 in glioma patients. *P < 0.05, **P < 0.01, ***P < 0.001. ****P < 0.0001

Notably, according to the classification of central nervous system tumours divided adult gliomas based on IDH status and 1p19q codeletion status [39], similar results from TCGA and CGGA databases were also determined that the highest mRNA expression of HK2 was found in GBM (Fig. 3A-B). With the higher histological grade of glioma, the mRNA expression of HK2 tended to be higher. Further, we used the Kaplan-Meier plotter to analyse the prognostic values of the mRNA expression of HK2 in new histological manifestations of glioma. The results from TCGA database showed that HK2 mRNA expression showed no correlation with the prognosis of Oligodendroglioma (*P* = 0.97). However, higher mRNA expression of HK2 was associated with poorer prognosis in Astrocytoma (*P* = 0.018) and glioblastoma (*P* = 0.026) (Fig. 3C-E). CGGA database shows the same results. The mRNA expression of HK2 also showed no correlation with the prognosis of Oligodendroglioma (*P* = 0.65). In contrast, the higher mRNA expression of HK2 was associated with poorer prognosis in Astrocytoma (*P* = 0.00074) and glioblastoma (*P* < 0.0001) (Fig. 3F-H).

Since previous studies have shown that IDH mutations are closely related to the prognosis of glioma [39], we next analysed the role of IDH1 and IDH2 mutations in glioma by using the GSCA database. The mutation rate of IDH1 (R132H) in LGG was 77.57%, and the mutation rate in glioblastoma was 6.45%. IDH2 (R172K) mutation rate in LGG was 3.99% and in glioblastoma was 0.25% (Fig. 3I-L). The results showed that IDH1 mutation was dominant in glioma compared with IDH2. Next, we analysed IDH1 mutation and its prognostic value in LGG and GBM patients. The results from Kaplan-Meier plot showed that IDH1 mutation was associated with better OS in LGG (Fig. 3M) and glioblastoma (Fig. 3N). Finally, we analysed the relationship between HK2 expression and IDH mutation. The results of TCGA and GSCA databases showed that compared with IDH mutation group, the level of HK2 gene expression was significantly higher in IDH wild type group (Fig. 3O-P).

Next, we subjected glioma TMA for immunostaining by applying anti-HK2 antibody (Fig. 3Q). The results revealed that the lower expression of HK2 was mainly determined in lower-grade gliomas (I and II; *n* = 40). In contrast, HK2 staining intensity was observed to be elevated in higher-grade gliomas (HGG; III and IV; *n* = 41), indicating that HK2 expression is positively correlated with the malignancy of glioma patients (Fig. 3R). Moreover, we correlated HK2 expression with clinicopathologic characteristics of glioma (Table 1). Correlation analysis revealed that HK2 expression was positively associated with high-grade gliomas (*P* < 0.001) and tumour recurrence (*P* < 0.001). There was no significant difference between HK2 expression and age (*P* = 0.735) and gender (*P* = 0.939). In addition, we then compared patients’ survival in relation to the HK2 expression in LGG and HGG cohorts. We found that the worse prognosis was positively related to the expression level of HK2 in glioma patients (Fig. 3S). Age, gender, grades and tumour recurrence were included in the Cox multivariate survival analysis (Table 2). The results showed that grades (*P* = 0.016) and tumour recurrence (*P* = 0.01) were risk factors for the prognosis of glioma patients. Together, our findings suggested that HK2 expression might be a potential biomarker for predicting prognosis of the patients with glioma.

**Table 1:** Correlation between HK2 expression and clinicopathologic characteristics of glioma patients

Characteristics	Cases	HK2 low expression	HK2 high expression	***P***-value
**Age (year)**				
≥ 60	15	8 (53.3%)	7 (46.7%)	0.735
< 60	66	32 (48.5%)	34 (51.5%)	
**Gender**				
Male	55	27 (49.1%)	28 (50.9%)	0.939
Female	26	13 (50%)	13 (50%)	
**Grades**				
LGG (I + II)	40	37 (92.5%)	3 (7.5%)	< 0.001
HGG (III + IV)	41	3 (7.3%)	38 (92.7%)	
**Tumor recurrence**				
Yes	47	15 (36.2%)	32 (63.8%)	
No	34	25 (73.5%)	9 (26.5%)	< 0.001

**Table 2:** Multivariate Cox regression analyses for the various prognostic parameters in patients

Prognostic variables	***P***-value	Hazard Ratio	95% confidence interval
**Age**	0.341	1.013	(0.9861–1.042)
**Gender**	0.364	0.684	(0.301–1.553)
**Grades**	0.016	3.620	(1.274–10.284)
**Tumor recurrence**	0.010	0.067	(0.009–0.518)

### The inhibition of HK2 expression suppresses tumour metastasis and VM formation in glioma cells

**Fig. 4:**
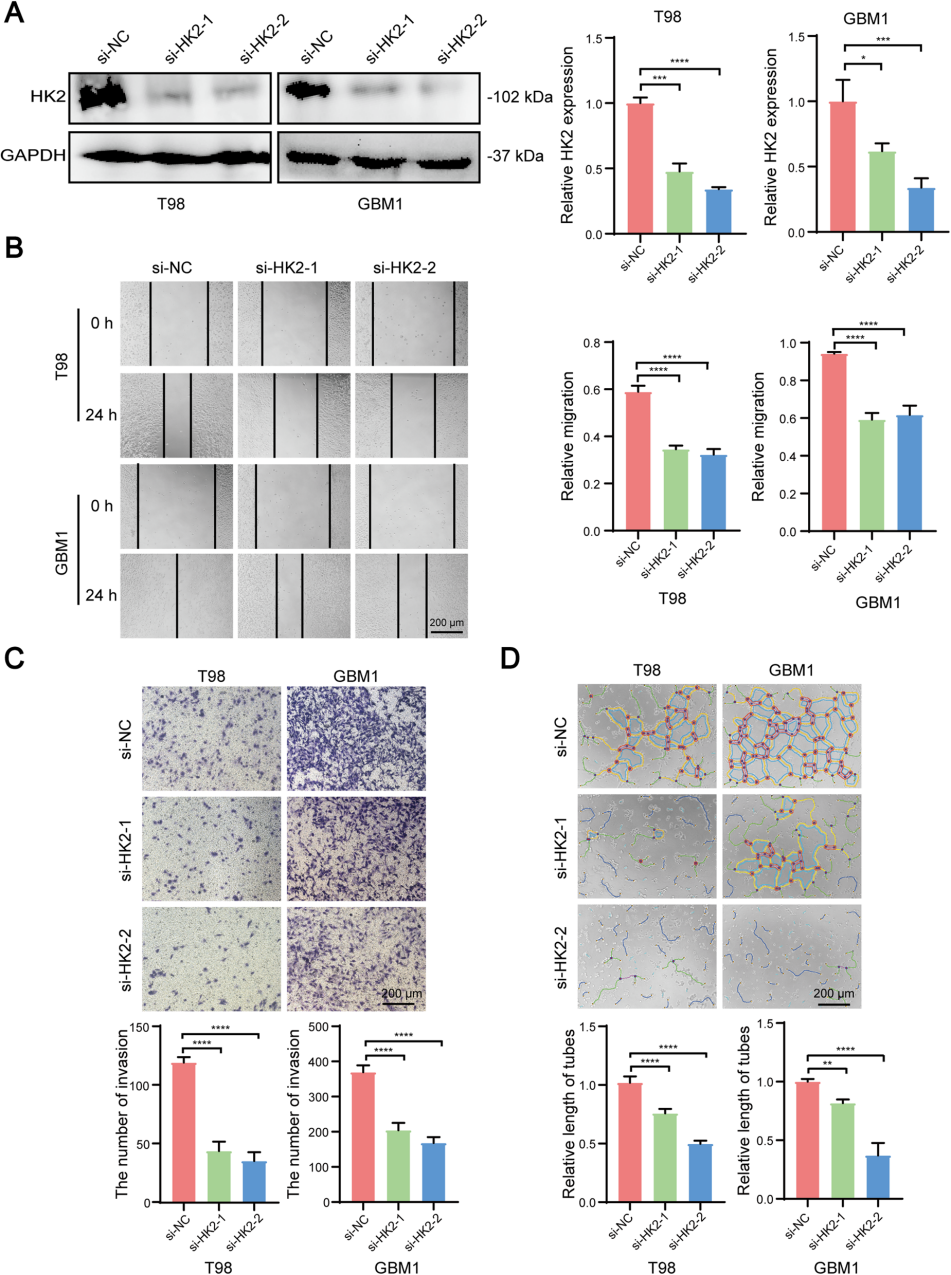
Knockdown of HK2 expression results in the suppression of tumour metastasis and VM Formation in glioma cells. **A** Representative images and quantification of the inhibition of HK2 expression in si-RNAs targeted HK2 (si-HK2–1 and si-HK2–2)-mediated HK2 knockdown in T98 and GBM1 cell lines. **B**-**C** The suppression of HK2 expression can inhibit tumour metastasis by using wound healing (**B**) and transwell (**C**) assays in T98 and GBM1 cell lines. **D** Tube formation assay shows HK2 knockdown could induce the inhibition of VM Formation in glioma cells. Bars, 200 μm. Data shown as mean ± SEM.*P < 0.05, **P < 0.01, ***P < 0.001, ****P < 0.0001

Since the aberrant HK2 expression was critical for glioma development in vivo and in vitro, we next examined whether the inhibition of HK2 could suppress tumour development in glioma cells. Therefore, we attempted to establish HK2-knockdown glioma cell lines through respectively transfecting two siRNAs targeted to HK2 in T98 and GBM1 cells. Western blot assay indicated that the transfection of either si-HK2 miRNA (si-HK2–1 and si-HK2–2) could efficiently inhibit HK2 expression in these two cell lines (Fig. 4A). Further wound healing and transwell assays revealed that the metastatic capabilities were significantly inhibited in HK2-silenced T98 and GBM1 cells (Fig. 4B-C). In addition, we also found that the VM Formation was also inhibited after inhibiting HK2 expression in glioma cells (Fig. 4D), suggesting that the HK2 is potentially related to tumorigenesis and tumour development in the patients with glioma.

### HK2 is involved in glioma development through regulating immune infiltration and immune checkpoints

**Fig. 5:**
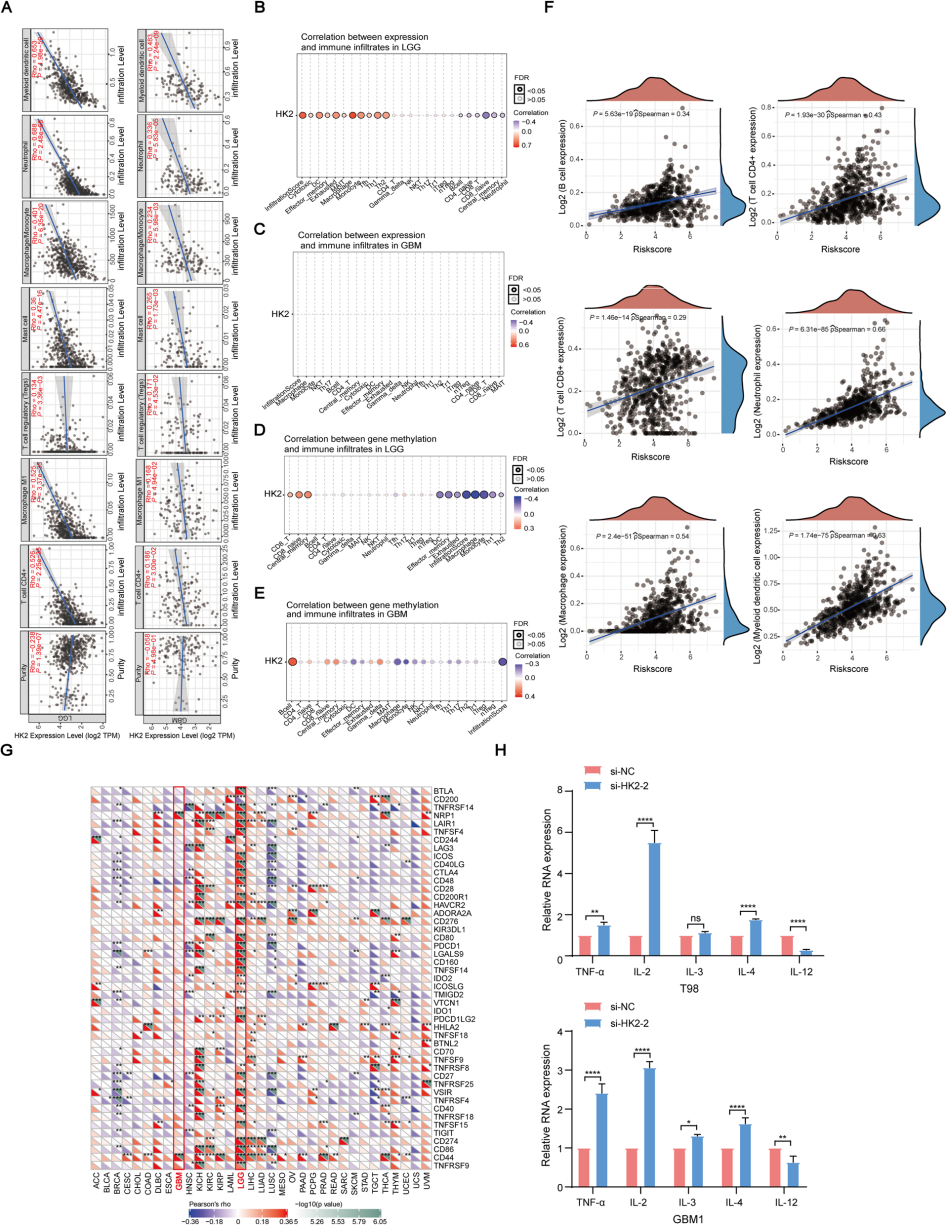
The correlation between HK2 expression and immune infiltration level in LGG and GBM. **A** Evaluation of the correlation between HK2 expression and immune infiltration by using the TIMER 2.0 database. **B**-**E** The correlation between the expression (**B**-**C**) or DNA methylation (**D**-**E**) of HK2 expression and immune infiltration in LGG and GBM specimens from the GSCA database. **F** The correlation between the expression of HK2 and immune score in gliomas. **G** The correlation between HK2 and the confirmed immune checkpoints in gliomas. **H** The mRNA levels of TNF-α, IL-2, IL-3, IL-4, and IL-12 in HK2-knockdown and control groups in T98 and GBM1 cell lines. Data are shown as mean ± SEM. *P < 0.05, **P < 0.01, ****P < 0.0001, ns, not significant

The TIMER2.0 database-based analyses illustrated that HK2 expression may be associated with immune infiltration, indicating the potential prognostic role of HK2 in glioma patients. In LGG and GMB specimens, HK2 expression was related to infiltration of CD4+ T cells, M1 macrophages, regulatory T cells (Tregs), mast cells, macrophages/monocytes, neutrophils and myeloid dendritic cells (Fig. 5A). Specifically, in LGG specimens, the analysis of the GSCA database revealed that HK2 expression was positively correlated with the infiltration score, cytotoxicity, dendritic cells (DCs), macrophages, monocytes, and T helper 1 and 2 (Th1/2) cells but negatively correlated with B cells, naïve CD4 T cells (CD4_naïve), naïve CD8 T cells (CD8_naïve), CD8 T cells (CD8_T), central memory and neutrophils. Additionally, we found positive correlations between HK2 expression and the infiltration score, macrophage, monocyte, natural killer T cells (NKT), T-helper 17 (Th17) cells, and B cells and negative correlations between the nTreg, CD4_naïve, CD8_naïve, and CD8_T groups in GBM patients (Fig. 5B-C). In addition, CD8_T, CD8_naïve, central memory, and B cells were found to be associated with the DNA methylation of HK2 in both LGG and GBM groups (*FDR* < 0.05) (Fig. 5D-E).

We then evaluated the correlation between HK2 expression and immune score by using the TCGA dataset. The ESTIMATE algorithm revealed that HK2 expression was positively related to immune invasion in glioma specimens (Fig. 5F). Interestingly, we found that immune checkpoints were also involved in this process (Fig. 5G). TISIDB database analysis showed a positive correlation between HK2 expression and DNA methylation in the LGG and GBM groups with immune stimulators, immune inhibitors or MHC molecules (Supplementary Fig. S3A-F). Therefore, to further confirm our hypothesis, we next examined the expression of several key inflammatories and immune modulators, including TNF-α, IL-2, IL-3, IL-4, and IL-12, in HK2-silenced T98 and GBM1 glioma cells. qRT-PCR assay demonstrated the upregulation of TNF-α, IL-2, IL-3, and IL-4 expression, whereas the downregulation of IL-12 expression in HK2-knockdown glioma cells (Fig. 5H), suggesting that HK2 is essential for glioma development through regulating immune infiltration.

### Identification and analysis of genes related to HK2 expression in glioma

**Fig. 6:**
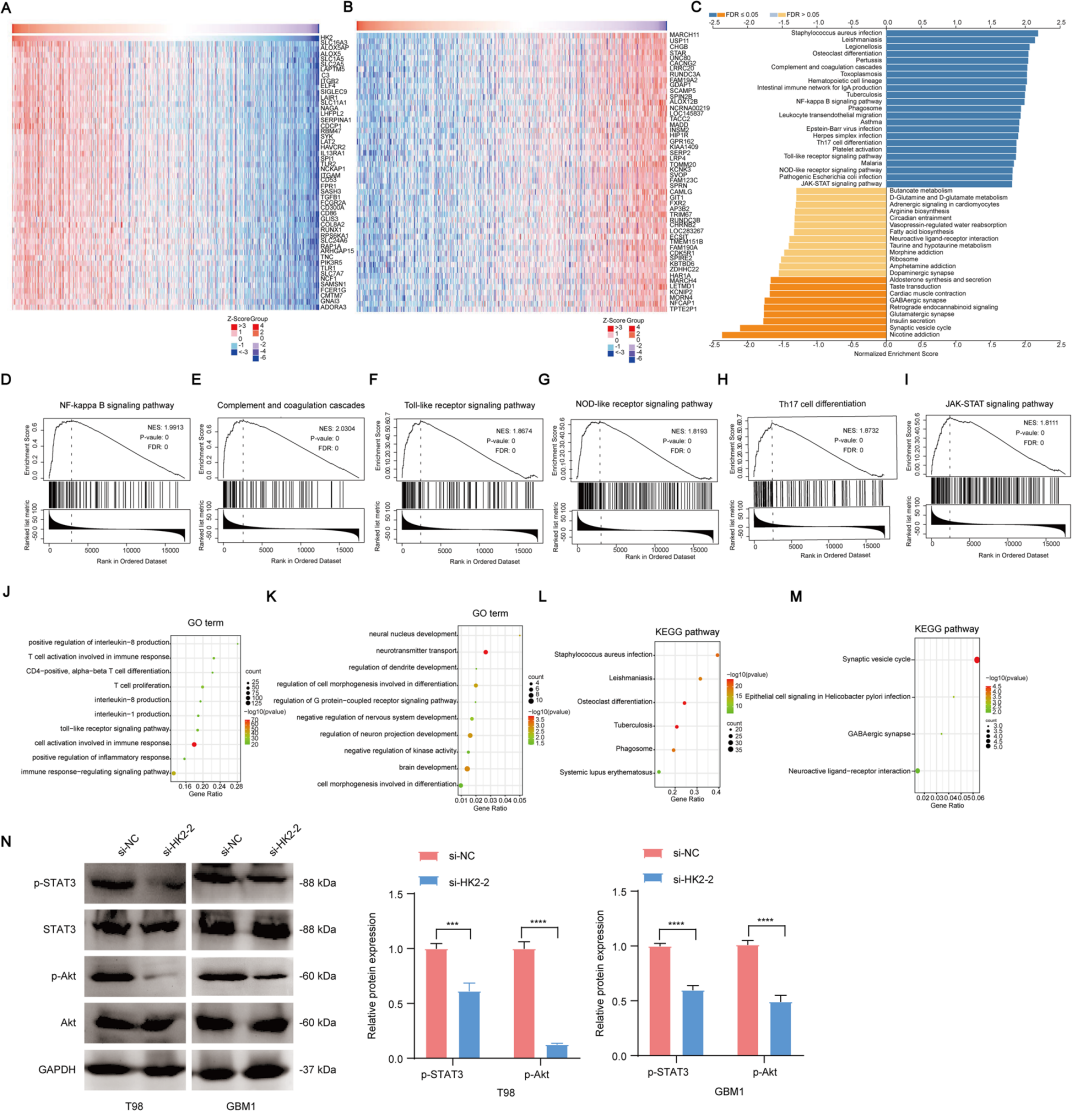
GO term, KEGG pathway, and GSEA enrichment analyses of HK2-related genes. **A**-**B** Heatmap of the top 50 positively and negatively related genes with HK2. **C** KEGG pathway enrichment analysis using the GSEA database. **D**-**I** Gene set enrichment plots of the NF-κB (**D**), complement and coagulation cascades (**E**), Toll-like receptor (**F**), NOD-like receptor (**G**), Th17 cell differentiation (**H**), and JAK-STAT (**I**) signalling pathways. **J**-**M** GO and KEGG enrichment analyses of HK2 positively or negatively related genes (Pearson’s rho ≥0.5, *P* < 0.05). **N** Expression levels of p-STAT3, STAT3, p-Akt, Akt in HK2-knockdown and control groups in T98 and GBM1 cell lines**.** Data shown as mean ± SEM. *P < 0.05, **P < 0.01, ***P < 0.001, ****P < 0.0001

Due to the potential roles of HK2 in glioma development, we hypothesized that HK2 might represent a new target for the treatment of glioma. We identified the gene expression related to HK2 using the LinkedOmics database. The most significantly positively or negatively related genes (*n* = 50) and signalling pathways (*n* = 32), which were regulated by HK2, were identified by using heatmaps (Fig. 6A-B, Supplementary Table S6) and KEGG pathway enrichment analysis (Fig. 6C, Supplementary Table S7). Specifically, eukaryotic orthologous groups (KOG) identified the top six immune-related signalling pathways, including NF-κB, complement and coagulation cascades, Toll-like receptor, NOD-like receptor, Th17 cell differentiation, and JAK-STAT signalling pathways, which are regulated by HK2 (Fig. 6D-I).

Next, GO and KEGG cluster analyses were performed to identify potential HK2-regulated downstream signalling (|Pearson’s rho| ≥ 0.5, *P* < 0.05). GO term enrichment indicated that positively correlated HK2-related genes enriched in positive regulation of interleukin− 8 production, T cell activation involved in immune response, CD4-positive, alpha-beta T cell differentiation, T cell proliferation, interleukin− 8 production, nterleukin-1 production, toll−like receptor signalling pathway, cell activation involved in immune response, positive regulation of inflammatory response and immune response−regulating signalling pathways, were positively regulated (Fig. 6J). In contrast, negatively correlated HK2-related genes were enriched in neural nucleus development, neurotransmitter transport, regulation of dendrite development, regulation of cell morphogenesis involved in differentiation, regulation of G protein−coupled receptor signalling pathway, negative regulation of nervous system development, regulation of neuron projection development, negative regulation of kinase activity, brain development and cell morphogenesis involved in differentiation (Fig. 6K). In addition, KEGG pathway analysis revealed that *Staphylococcus aureus* infection, leishmaniasis, osteoclast differentiation, tuberculosis, phagosome and systemic lupus erythematosus, synaptic vesicle cycle, epithelial cell signalling in *Helicobacter pylori* infection, GABAergic synapse and neuroactive ligand−receptor interaction signals were involved in HK2-associated pathways (Fig. 6L-M). Moreover, it is noted that similar results were also determined by using western blot analyses that the inactivation of PI3K/Akt signalling pathways after HK2 knockdown in T98 and GBM1 glioma cells (Fig. 6N).

### The mRNA levels of hub genes of HK2-related genes might be independent prognostic biomarkers in glioma patients

**Fig. 7:**
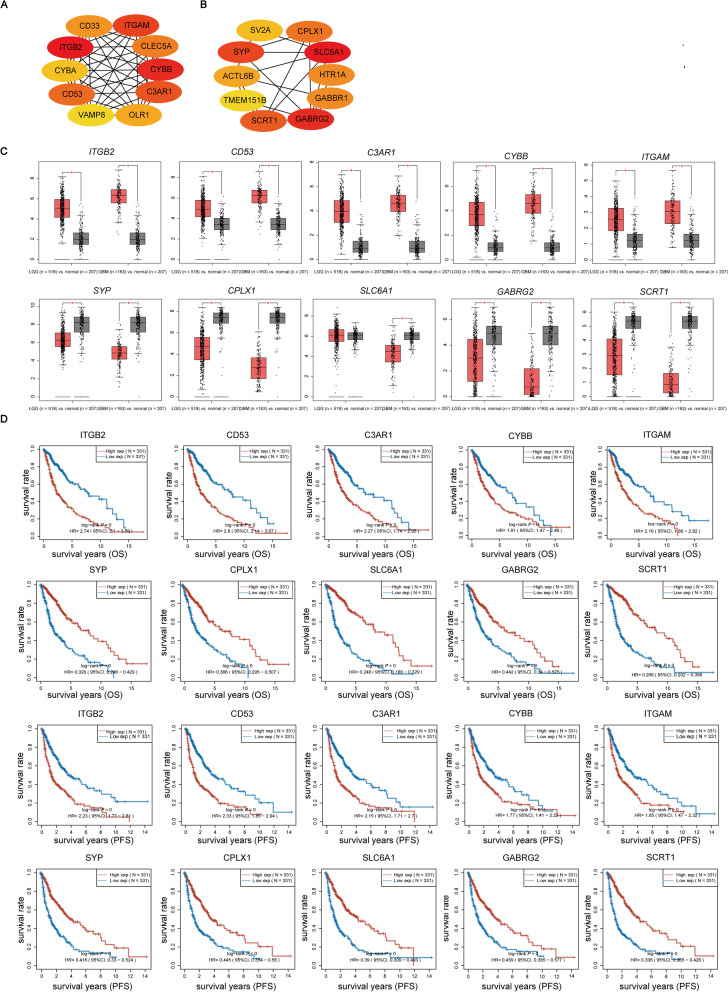
Graphic representation of hub genes of the PPI network and the correlation between the top 10 hub genes and the prognostic prediction in glioma patients. **A**-**B** The interactive network of the top 10 hub genes in HK2 positive correlation genes and top 10 hub genes in HK2 negative correlation genes by using the algorithm. **C** The expression of the top 10 hub genes positively and negatively correlated with HK2 in LGG and GBM specimens. **D** The correlation between the expression of these hub genes and the prognosis (OS and FPS) of glioma patients. *P < 0.05

The STRING database showed an interactive network of most genes with strong correlations to HK2 expression (|Pearson’s rho| ≥ 0.5, *P* < 0.05) (Supplementary Fig. S4A-B). Notably, the interactive network of the top 10 hub genes (Supplementary Tables S8 & S9) was analysed by the CytoHubba plug-in (Fig. 7A-B). The results showed that the top 5 positive HK2-related hub genes were *ITGB2, CD53, C3AR1, CYBB and ITGAM,* whereas the top 5 negative HK2-related hub genes were *SYP, CPLX1, SLC6A1, GABRG2 and SCRT1*. GEPIA analysis then confirmed that the expression of all these hub genes was identically regulated in LGG and GBM specimens, except for *SLC6A1* expression, which was not significantly different in the LGG group (Fig. 7C). In addition, the GEPIA database also showed correlations between the individual expression of these hub genes and HK2 (Supplementary Fig. S5A-J), suggesting that HK2 is probably involved in glioma development by regulating its hub genes.

We also found that the high expression of the top 5 positively HK2-related hub genes was associated with poorer OS and PFS, whereas the negative HK2-related hub genes showed better prognosis in an expression level-dependent manner in gliomas (Fig. 7D), suggesting that the expression of these hub genes was involved in the prognosis of glioma patients. In addition, univariate Cox regression analyses indicated significant correlations between the expression of these hub genes (*ITGB2, CD53, C3AR1, CYBB, ITGAM, SYP, CPLX1, GABRG2, SLC6A1* and *SCRT1*) and the prognosis of glioma (Supplementary Fig. S6A-B). Moreover, further multivariate Cox regression analysis revealed that the expression of *CYBB* (*P* = 0.00087), *CD53* (*P* = 0.04883), *SCRT1* (*P* = 0.00071) and *SYP* (*P* = 0.02491) can be independent factors of the prognosis of gliomas (Supplementary Fig. S6C-D) and predict the survival of glioma patients (Supplementary Fig. S6E-F).

**Fig. 8:**
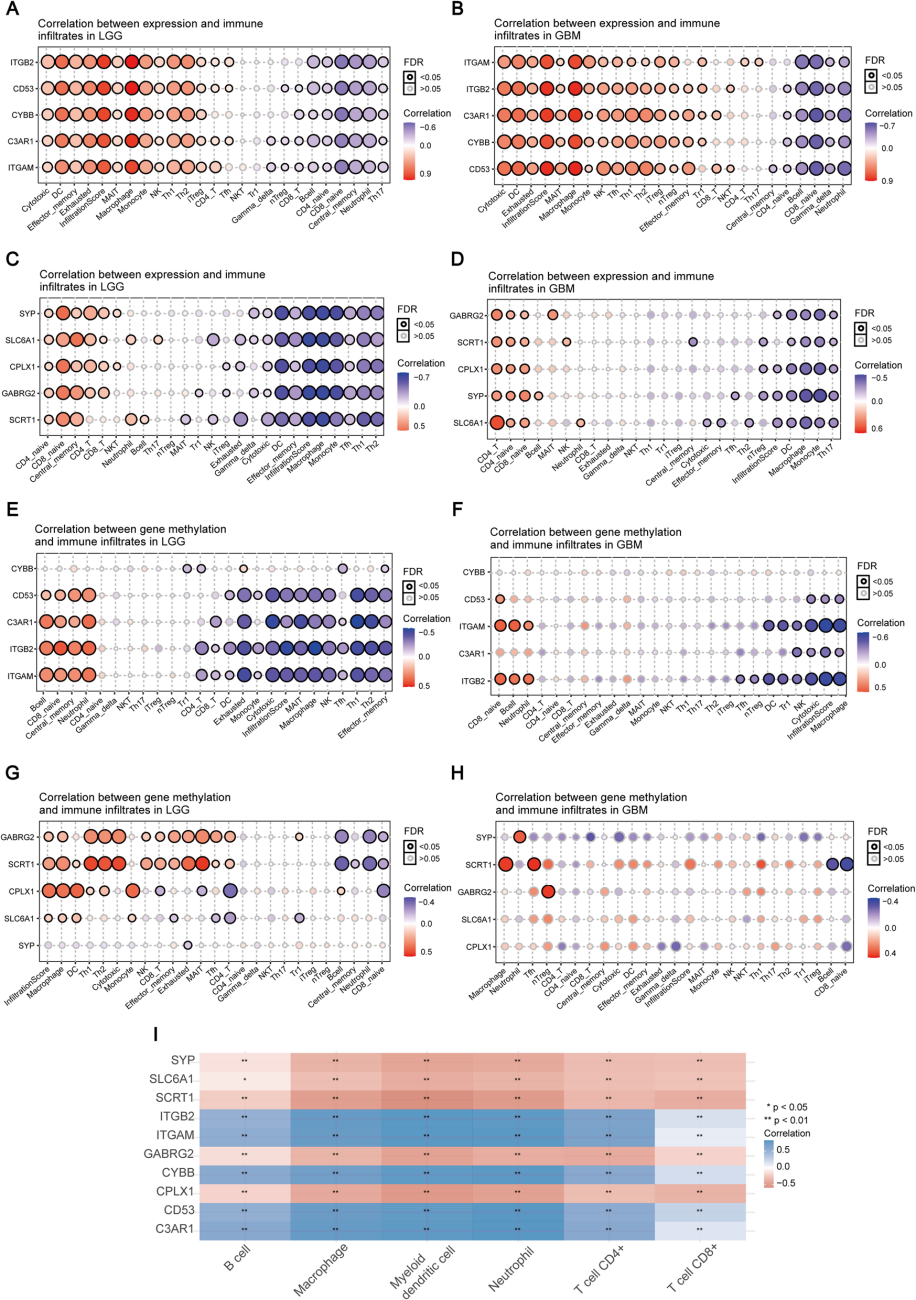
Correlations between hub genes expression or DNA methylation and immune cells. **A**-**H** The correlation between the expression (**A-D**) or DNA methylation (**E**-**H**) of all these hub genes and immune infiltration in LGG and GBM specimens. **I** The correlation between the expression of hub genes and immune score in glioma. *P < 0.05, **P < 0.01

We subsequently evaluated the correlation between the expression of these hub genes and immune infiltration. Our results indicated that the expression or DNA methylation of all these HK2-related hub genes is closely correlated with immune cell subtypes (Fig. 8A-H). In addition, we also found a correlation between hub genes and immune score in glioma specimens (Fig. 8I), suggesting that the expression of hub genes plays a role in glioma development by regulating immune infiltration.

## Discussion

HK2 is usually expressed in skeletal, cardiac muscle and adipose tissues [40], as well as many malignant tumour tissues [41]. Recent study indicated that the regulation of cancer cell metabolism may be partially induced by the recruitment of the immune and inflammatory cells [42]. In addition, the metabolic reprogramming is not only closely associated with tumorigenesis, angiogenesis, tumour invasion and migration, but also involved in the process of proliferation, differentiation, and execution of effector functions, which is important to the immune response [43–45]. In the tumour microenvironment, the aberrant or intermediates metabolites of metabolism in cancer cells could be associated with the regulation of activation, differentiation, and function of immune cells [46, 47]. A very recent study indicated that the cancer cells are able to reduce the metabolic fitness of tumour-infiltrating immune cells, and subsequently escape anti-tumour immune response [48, 49]. Therefore, the overlapping immune response and metabolic reprogramming in cancer cells would be critical for anti-tumour immune response in cancer. In the present study, we found that HK2 is highly expressed in gliomas and that HK2 expression is positively correlated with OS, PFS, and DSS in glioma patients, suggesting that it may be a potential clinical target for the treatment of glioma. Our results also indicated that HK2 expression is associated with recognized immune checkpoints and the abundance of multiple immune infiltrates, including CD4+ T cells, M1 macrophages, regulatory T cells, mast cells, monocytes, neutrophils and myeloid dendritic cells, in LGG and GBM specimens. There was a slight difference in the TIMER 2.0 and GSCA databases, which might result from different algorithms of different databases. Multiple database analyses can provide more comprehensive and reliable evidence in this study. Our results also demonstrated that the high expression of HK2 is associated with poor prognosis of glioma. We expected that HK2 may regulate innate immune cells and abnormally activate the immune response, resulting in the poor prognosis of gliomas. However, the underlying mechanism needs to be further investigated. Moreover, although there are certain correlations between HK2 expression and immune stimulators, immune inhibitors and MHC molecules in LGG and GBM patients, the underlying mechanism by which HK2 activates immune cells still needs to be further investigated.

A previous study indicated that PD-L1 can enhance glycolysis by regulating HK2 expression in non-small cell lung cancer (NSCLC), indicating that HK2 may play a role in tumour immunity [50], which is consistent with our results also showing that the HK2-regulated genes were mainly enriched in immune-related signalling pathways and the activation of immune cells (Fig. 6C-I). Further investigations revealed that the positive HK2-regulated genes were mainly associated with immune cells, the immune response and the secretion of inflammatory factors (Fig. 6J), suggesting that certain HK2-mediated immune response processes were involved in glioma development. The identification of the downstream signals of HK2 demonstrated that the high expression of hub genes, including *ITGB2, CD53, C3AR1, CYBB and ITGAM*, maybe a potential target for the treatment of glioma. In contrast, the upregulation of the top 5 negative HK2-regulated hub genes, such as *SYP, CPLX1, GABRG2, SLC6A1 and SCRT1*, was accompanied by a better prognosis of gliomas. Moreover, multivariate Cox regression revealed that *CYBB, CD53, SCRT1* and *SYP* might be independent factors for predicting the prognosis of gliomas. It is known that *CYBB* encodes the gp91-phox protein of phagocytic nicotinamide adenine dinucleotide phosphate (NADPH) oxidase [51]. Previous studies indicated that NADPH oxidase 2, also known as CYBB/NOX2, in conventional DCs (cDCs) regulates endocytosed MOG (myelin oligodendrocyte protein) antigen processing and supports MOG antigen presentation to CD4+ T cells through LC3-associated phagocytosis (LAP) [52, 53]. CD53 is a member of the tetraspanin superfamily expressed exclusively within the immune compartment. CD53 is highly expressed in a variety of immune cells, including B cells, CD4+ T cells, CD8+ T cells, dendritic cells, macrophages and natural killer cells [54]. SCRT1 participates in gene regulation, metabolic processes and immune responses as a tissue-specific transcription factor [55]. CTLA4-associated SYP could regulate the RAS pathway and T cell activation [56]. Notably, our results also showed that HK2 and its hub genes activate different immune cells by regulating the expression of different immune-related genes, thereby affecting the prognosis of gliomas, suggesting that HK2 and hub genes probably play important roles in different immune processes and may be potential targets for the treatment of glioma.

## Conclusions

In summary, integrated bioinformatics approaches indicated that HK2 expression may mediate immune infiltration to affect the prognosis of glioma patients and may be a potential prognostic biomarker. In addition, HK2 and its hub genes mediate the activation of immune cells and the regulation of immune-related factors and are involved in the prognosis of glioma patients. Therefore, our findings demonstrated that HK2 and its hub genes are significantly correlated with prognosis and immune infiltration in glioma and may be potential targets for the treatment of patients with glioma.

## Supplementary Information

**Additional file 1: Fig. S1.** An overview of the proposed workflow.

**Additional file 2: Fig. S2.** GCCA database was used to evaluate HK2 gene expression with box plots according to clinical parameters in glioma patients. **A-I** The respective correlation between HK2 expression and histology (**A**), grade (**B**), IDH mutation status (**C**), 1p/19q co-deletion status (**D**), IDH mutation status & 1p/19q co-deletion status (**E**), 1p/19q co-deletion status in different grades (**F**), gender (**G**), age status (**H**) and progression status (**I**). *P < 0.05, **P < 0.01, ***P < 0.001.

**Additional file 3: Fig. S3.** The respective correlation between HK2 expression or methylation and immune stimulators, immune inhibitors, and MHC molecules in LGG and GBM specimens. **A-F** The respective correlation between HK2 expression and immunostimulatory (**A-B**), immunoinhibitory (**C-D**), and MHC molecules (**E-F**) is shown in LGG and GBM specimens. *P < 0.05, **P < 0.01, ***P < 0.001.

**Additional file 4: Fig. S4.** Construction of the PPI network using HK2 positively **A** and negatively **B** related genes (Pearson’s rho ≥ 0.5, *P < 0.05).

**Additional file 5: Fig. S5.** The correlation between the expression of HK2 and hub genes. **A-J**. The hub genes Including *ITGB2*, *CD53*, *C3AR1*, *CYBB*, *ITGAM*, *SYP*, *CPLX1*, *SLC6A1*, *GABRG2*, and *SCRT1*, in the LGG and GBM groups. *P < 0.05, **P <0.01, ***P < 0.001.

**Additional file 6: Fig. S6.** The correlation between hub genes and the clinicopathological features of glioma. **A-D** Univariate (**A-B**) and multivariate (**C-D**) Cox regression showed that the top 5 positively and negatively HK2-related hub genes were associated with glioma prognosis. **E-F** A nomogram was constructed by *CYBB, CD53,* and grade (**E**), as well as *SCRT1*, *SYP*, and gender (**F**), for predicting the survival of glioma. HR, Hazard ratio; 95% CI, 95% confidence interval. *P < 0.05, **P < 0.01, ***P < 0.001.

**Additional file 7:.** The uncropped immunoblotting images of full-length blots.

**Additional file 8: Table S1.** List of primer sequences for qRT-PCR.

**Additional file 9: Table S2.** Different datasets to analyze HK2 expression in pathological classification of gliomas (ONCOMINE).

**Additional file 10: Table S3.** The information of clinicopathological characteristics of patient with gliomas.

**Additional file 11: Table S4.** Correlation between HK2 expression and clinicopathologic characteristics of glioma patients.

**Additional file 12: Table S5.** Univariate and multivariate analyses of various prognostics parameters in patients with glioma Cox-regression analysis.

**Additional file 13: Table S6.** The identification of the top 50 positively and negatively related genes with HK2.

**Additional file 14: Table S7.** KEGG pathway enrichment analysis of HK2 related genes.

**Additional file 15: Table S8.** The functional roles of HK2 positively related hub genes.

**Additional file 16: Table S9.** The functional roles of HK2 negatively related hub genes.

## Data Availability

Publicly available datasets were analysed in this study and can be found in UCSC Xena (http://xena.ucsc.edu/), the Cancer Genome Atlas (https://portal.gdc.cancer.gov/), and the CGGA database (http://www.CGGA.org.cn/).

## Abbreviations

HK2: Hexokinase 2
LGG: Lower Grade Glioma
GBM: Glioblastoma
VM: Vasculogenic Mimicry
HKs: Hexokinases
OS: Overall survival
PFS: Progression-free survival
DSS: Disease-free survival
DFS: Disease-free survival
ROC: receiver operating characteristic
PPI: Protein-protein interaction
GSEA: Geneset enrichment analysis
KEGG: Kyoto Encyclopedia of Genes and Genomes
GO: Gene ontology
MMR: Mismatch repair
si-HK2: siRNAs targeting HK2
BSA: Bovine Serum Albumin
TMA: Tissue microarray
IHC: Immunohistochemistry
DCs: Dendritic cells
FDR: False discovery rate
