# Supplementary figures and images for: The expression of Hexokinase 2 and its hub genes are correlated with the prognosis in glioma
Source: BMC Cancer. 2022 Aug 18;22:900. doi: 10.1186/s12885-022-10001-y (PMC9386956; doi:10.1186/s12885-022-10001-y)

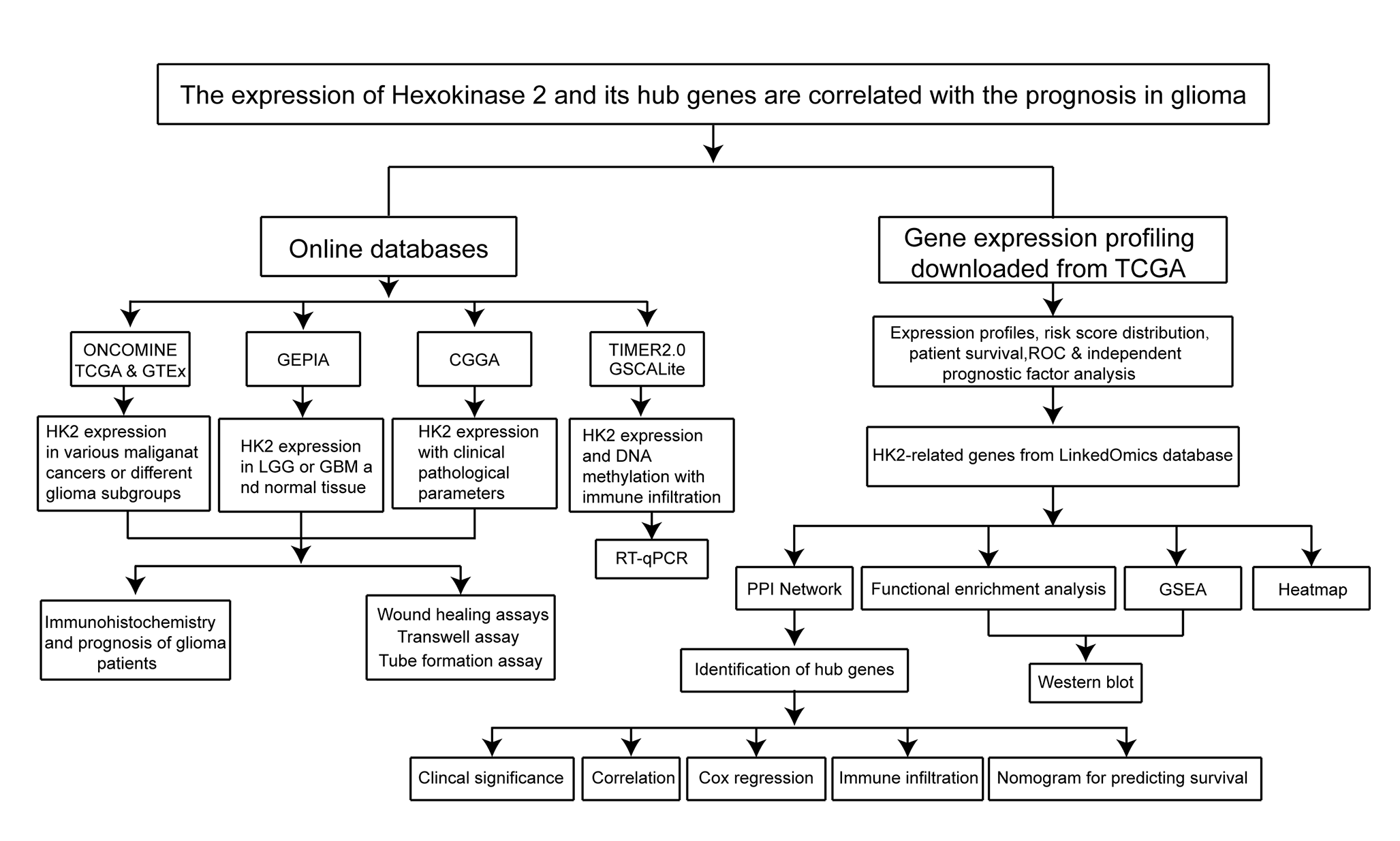

Supplement: Supplementary file 1 — Additional file 1: Fig. S1. An overview of the proposed workflow. [file 12885_2022_10001_MOESM1_ESM.tif]

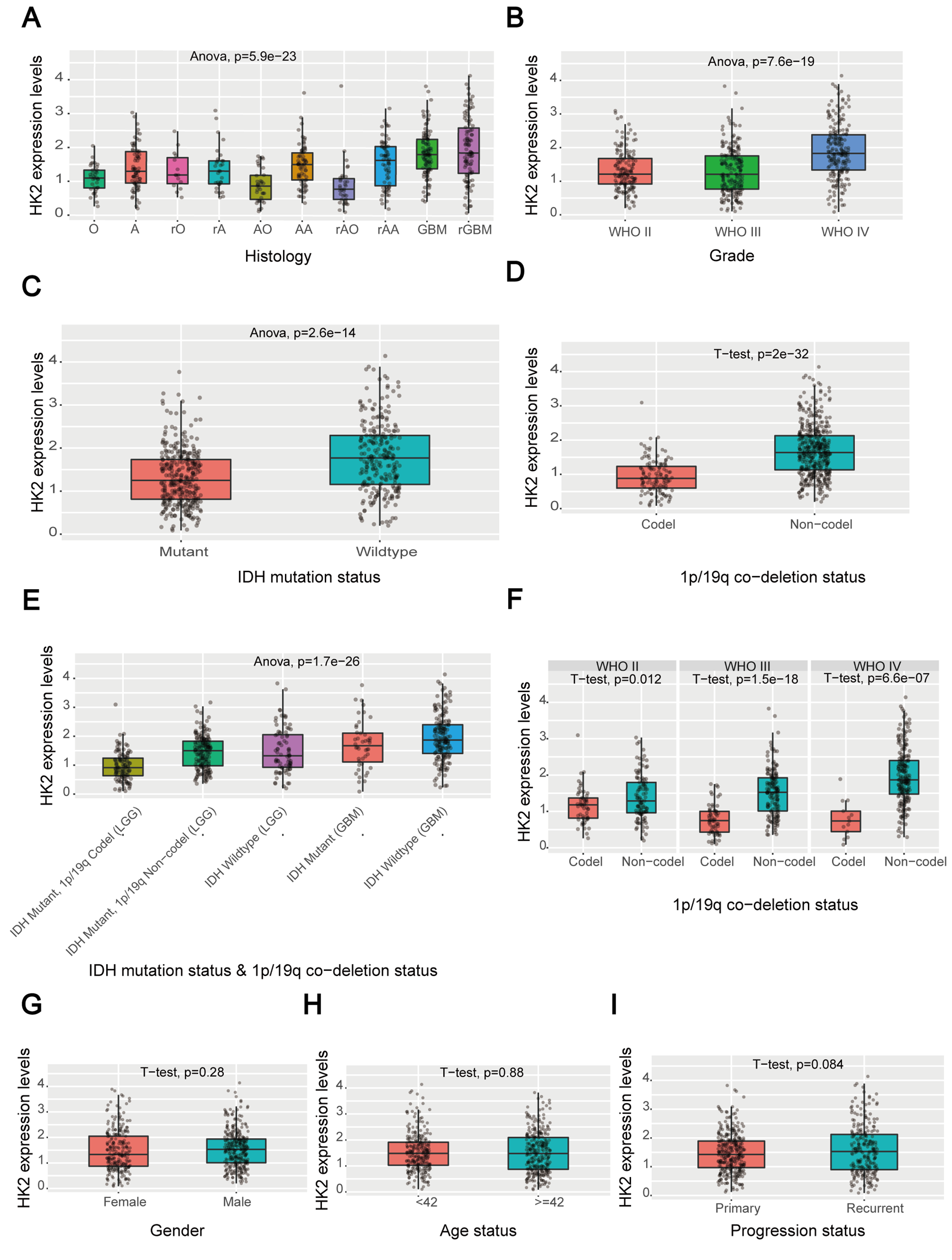

Supplement: Supplementary file 2 — Additional file 2: Fig. S2. GCCA database was used to evaluate HK2 gene expression with box plots according to clinical parameters in glioma patients. A-I The respective correlation between HK2 expression and histology (A), grade (B), IDH mutation status (C), 1p/19q co-deletion status (D), IDH mutation status & 1p/19q co-deletion status (E), 1p/19q co-deletion status in different grades (F), gender (G), age status (H) and progression status (I). *P < 0.05, **P < 0.01, ***P < 0.001. [file 12885_2022_10001_MOESM2_ESM.tif]

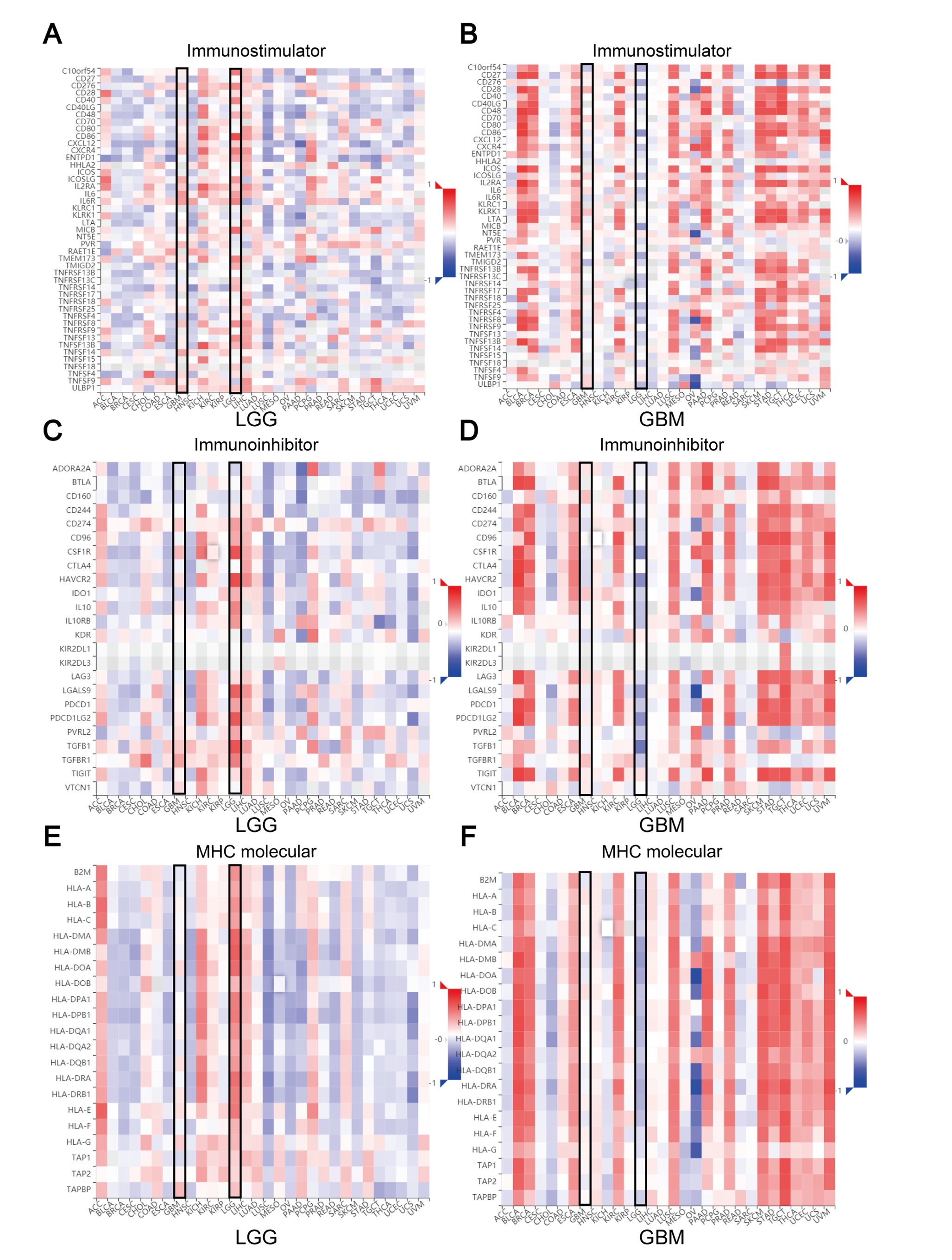

Supplement: Supplementary file 3 — Additional file 3: Fig. S3. The respective correlation between HK2 expression or methylation and immune stimulators, immune inhibitors, and MHC molecules in LGG and GBM specimens. A-F The respective correlation between HK2 expression and immunostimulatory (A-B), immunoinhibitory (C-D), and MHC molecules (E-F) is shown in LGG and GBM specimens. *P < 0.05, **P < 0.01, ***P < 0.001. [file 12885_2022_10001_MOESM3_ESM.tif]

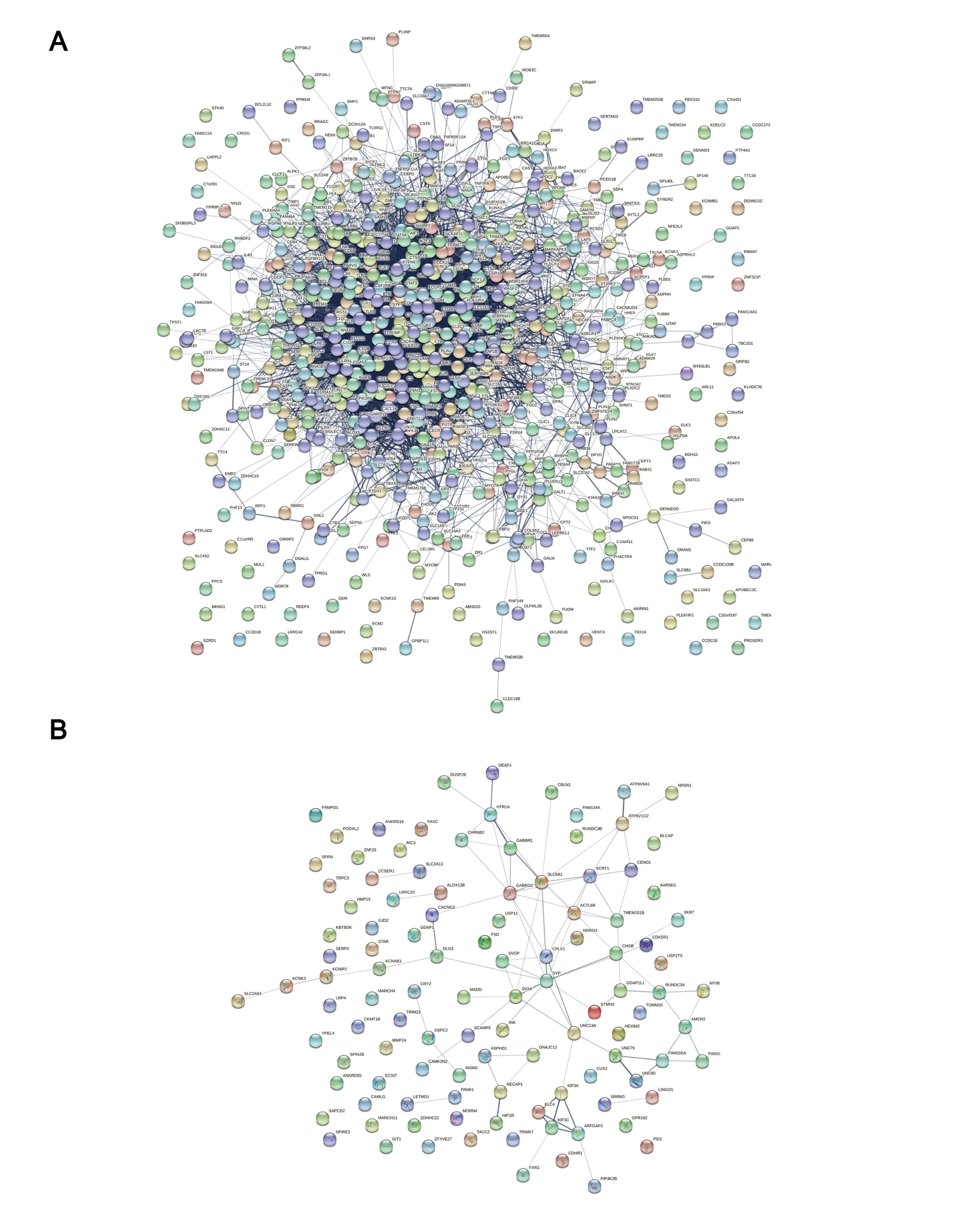

Supplement: Supplementary file 4 — Additional file 4: Fig. S4. Construction of the PPI network using HK2 positively A and negatively B related genes (Pearson’s rho ≥ 0.5, *P < 0.05). [file 12885_2022_10001_MOESM4_ESM.tif]

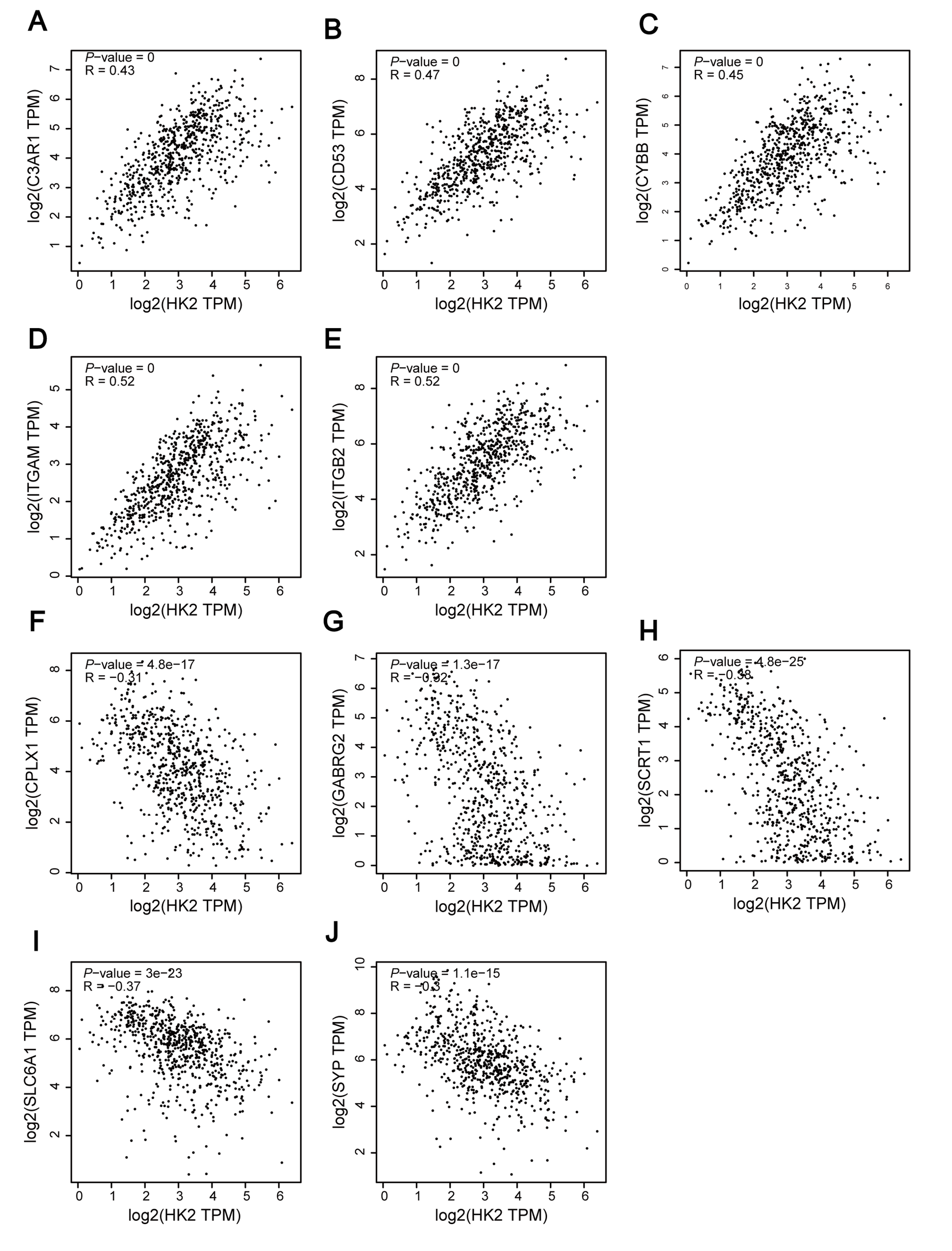

Supplement: Supplementary file 5 — Additional file 5: Fig. S5. The correlation between the expression of HK2 and hub genes. A-J. The hub genes Including ITGB2, CD53, C3AR1, CYBB, ITGAM, SYP, CPLX1, SLC6A1, GABRG2, and SCRT1, in the LGG and GBM groups. *P < 0.05, **P <0.01, ***P < 0.001. [file 12885_2022_10001_MOESM5_ESM.tif]

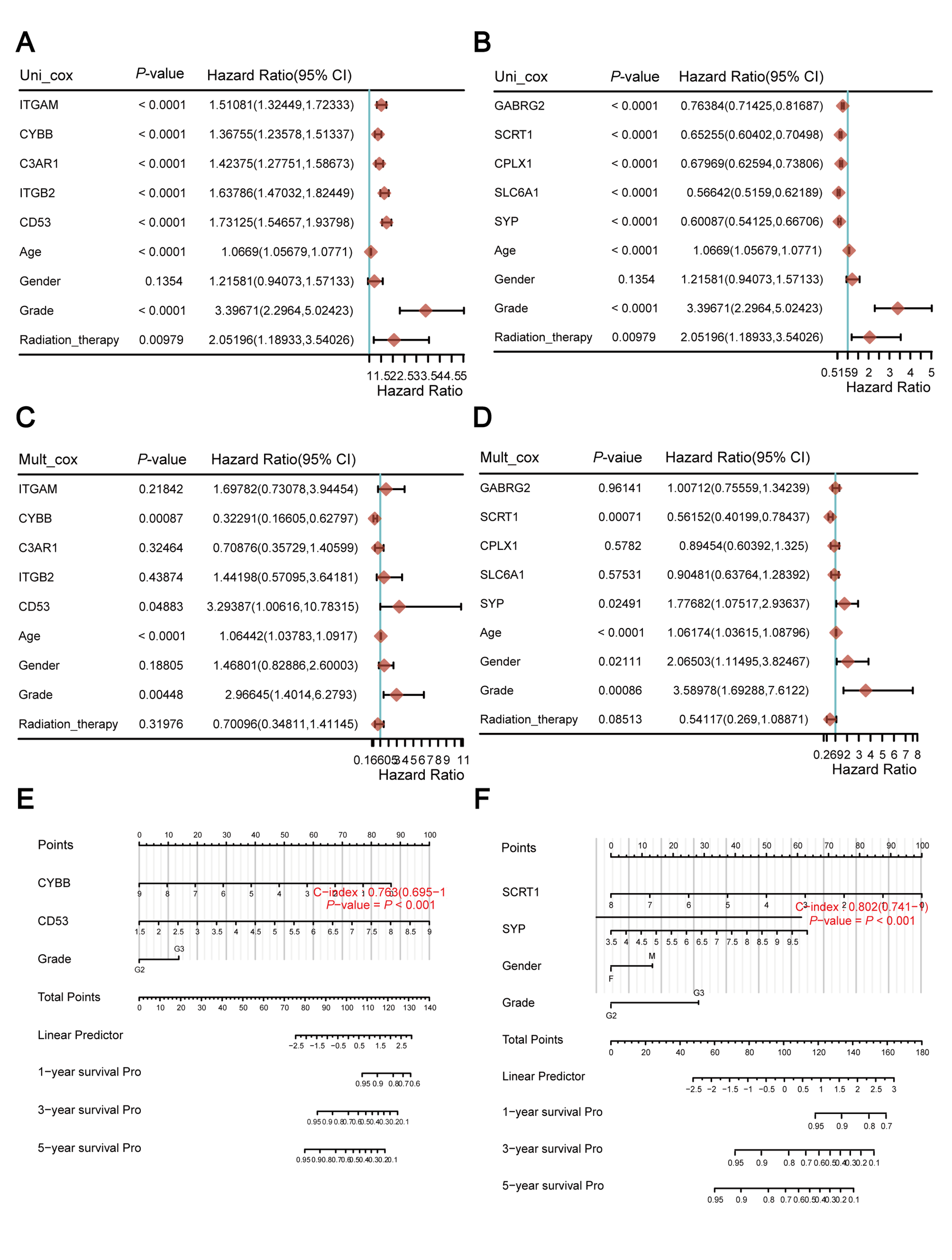

Supplement: Supplementary file 6 — Additional file 6: Fig. S6. The correlation between hub genes and the clinicopathological features of glioma. A-D Univariate (A-B) and multivariate (C-D) Cox regression showed that the top 5 positively and negatively HK2-related hub genes were associated with glioma prognosis. E-F A nomogram was constructed by CYBB, CD53, and grade (E), as well as SCRT1, SYP, and gender (F), for predicting the survival of glioma. HR, Hazard ratio; 95% CI, 95% confidence interval. *P < 0.05, **P < 0.01, ***P < 0.001. [file 12885_2022_10001_MOESM6_ESM.tif]

Figure 1

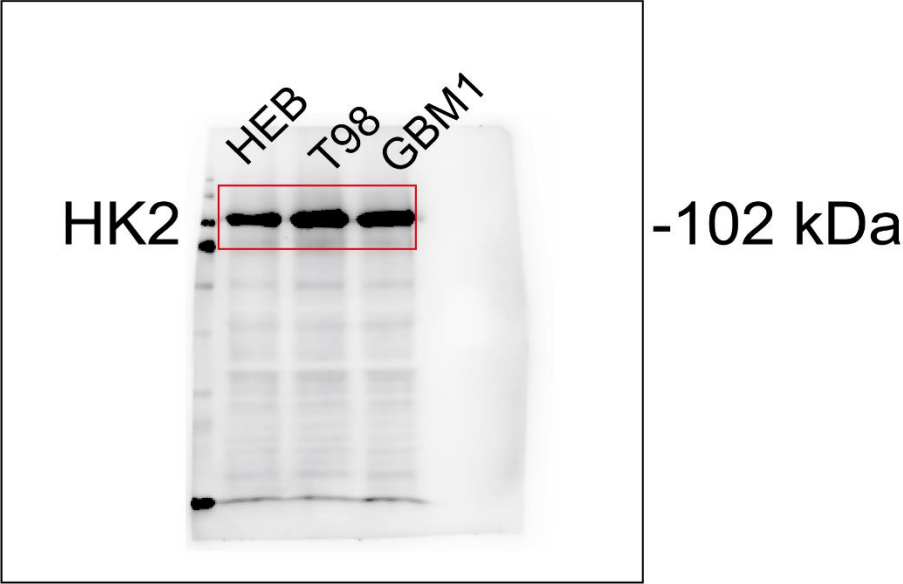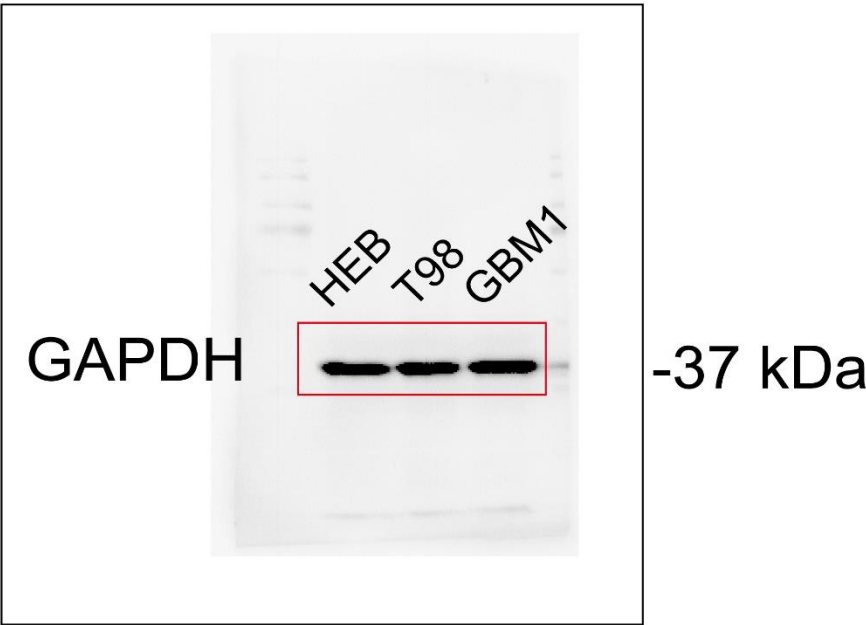

Figure 4

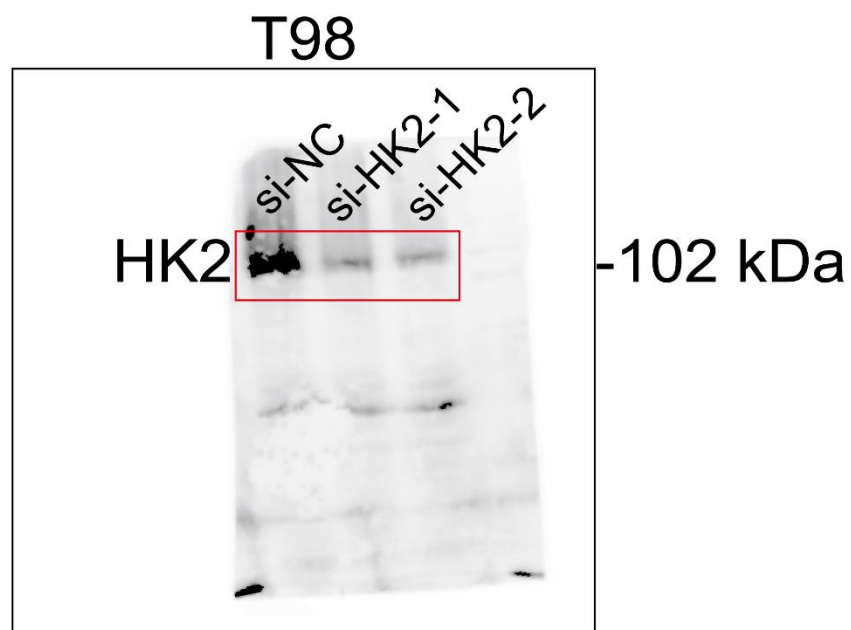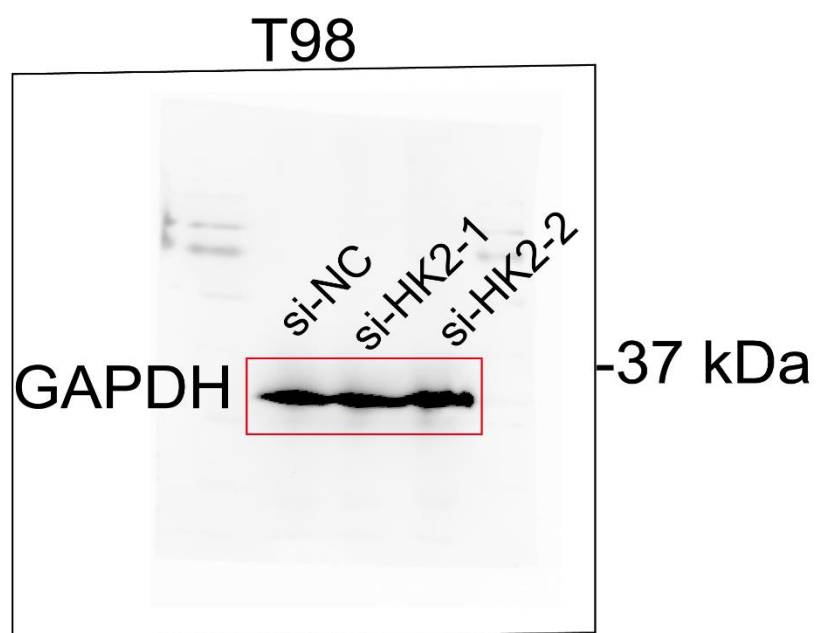

Figure 4

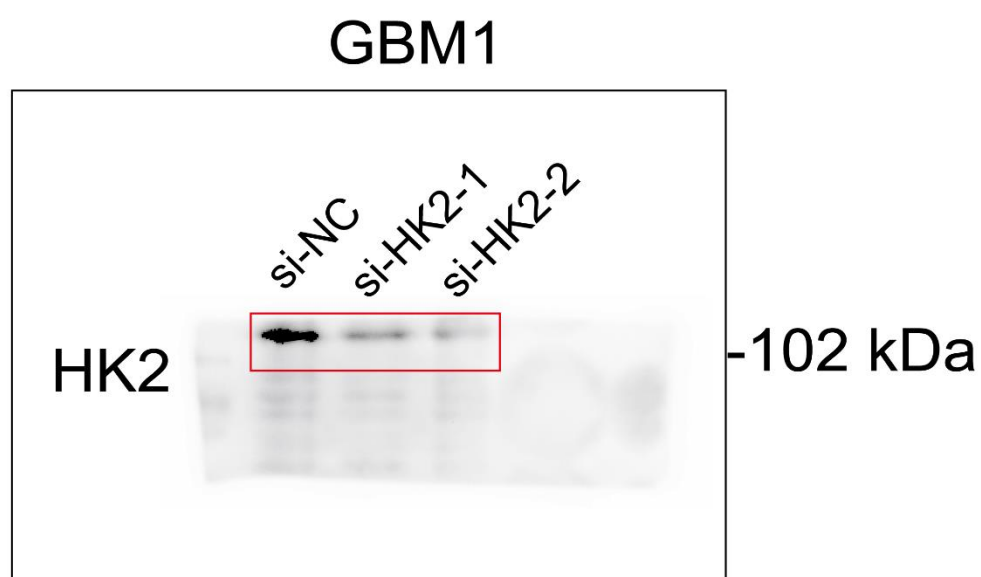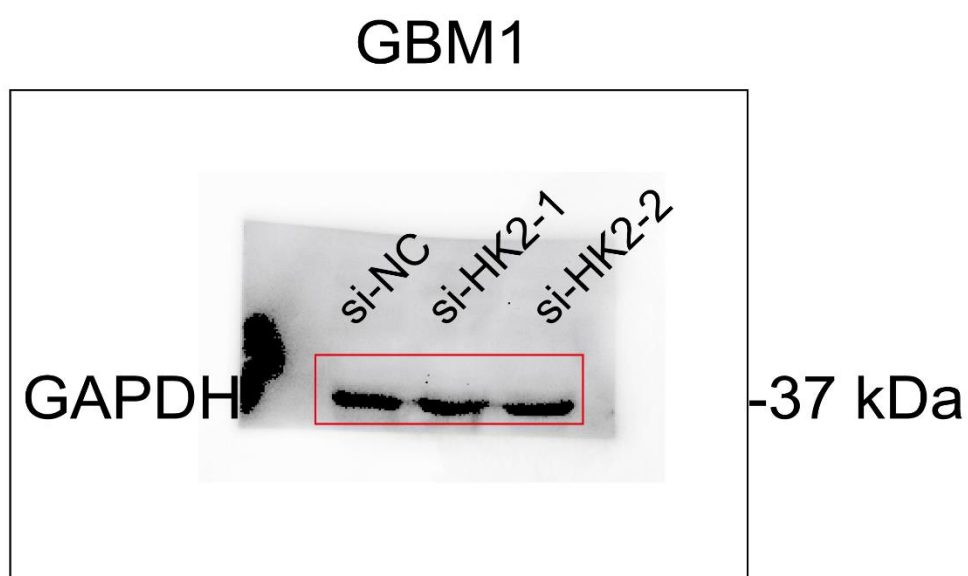

Figure 6

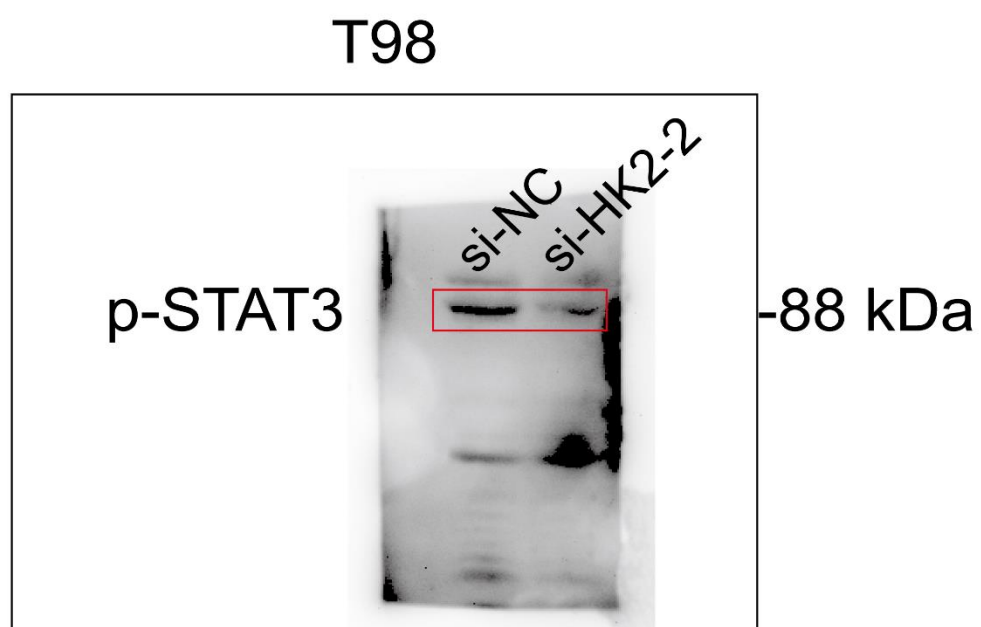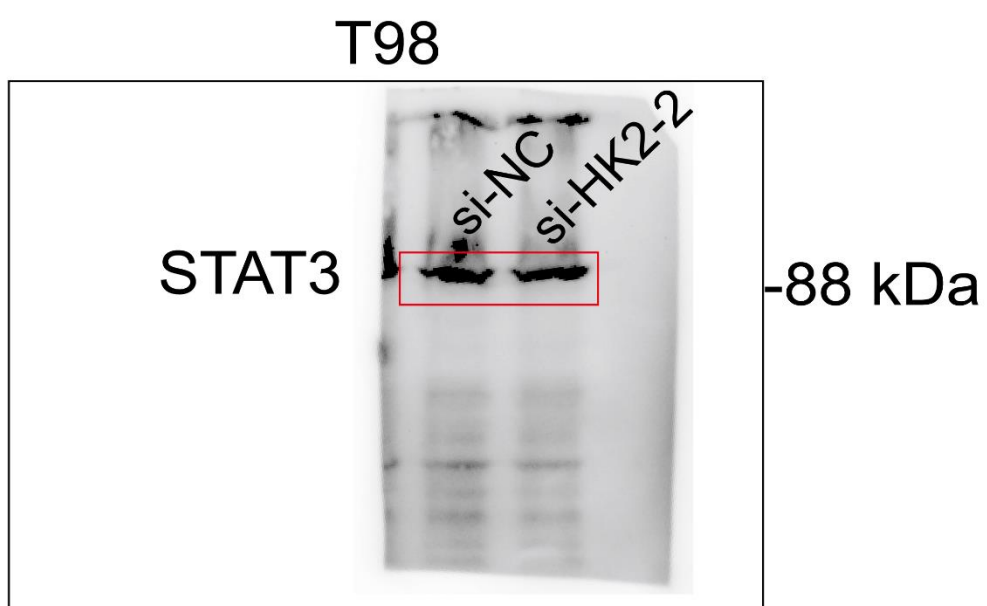

Figure 6

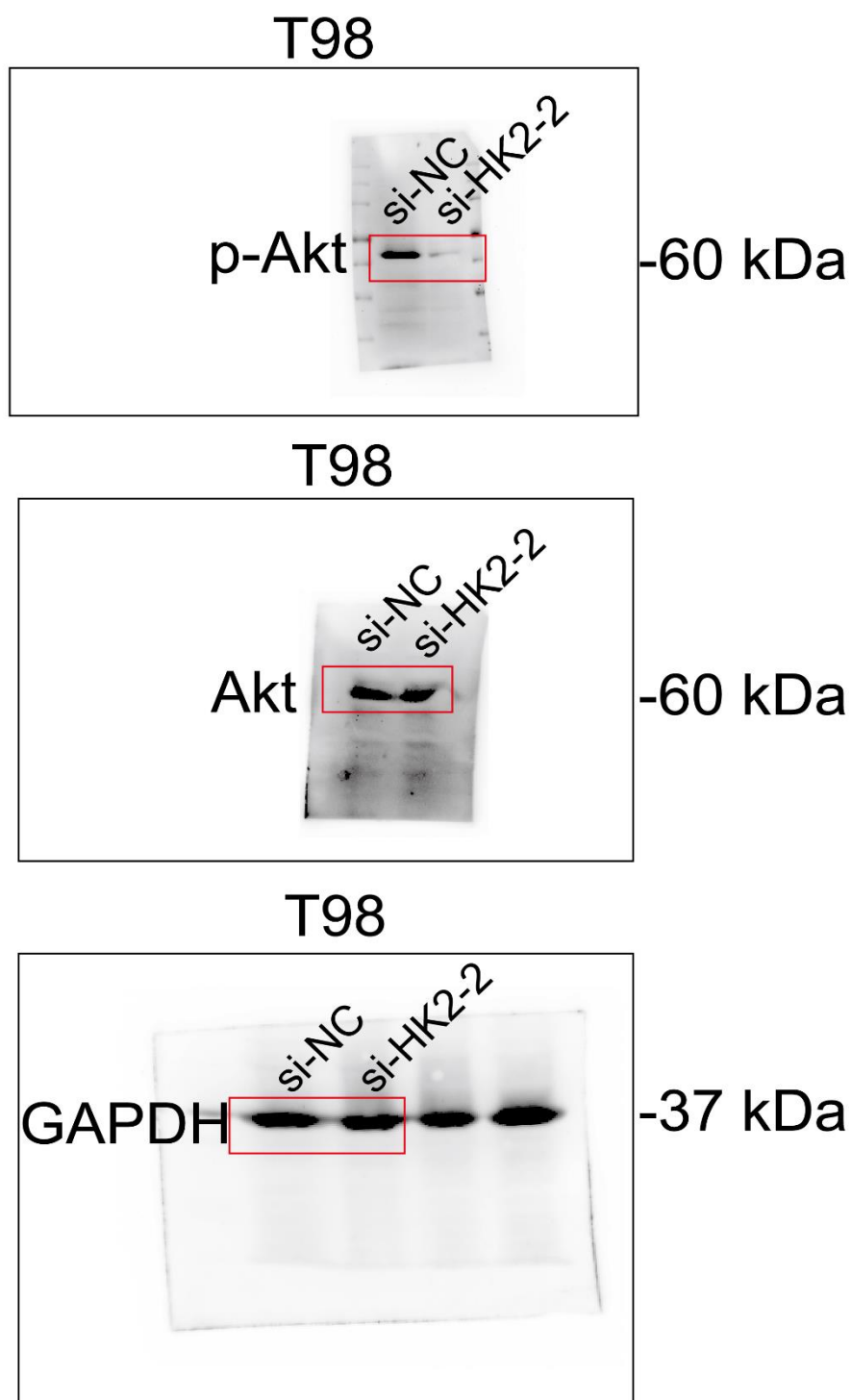

Figure 6

GBM1

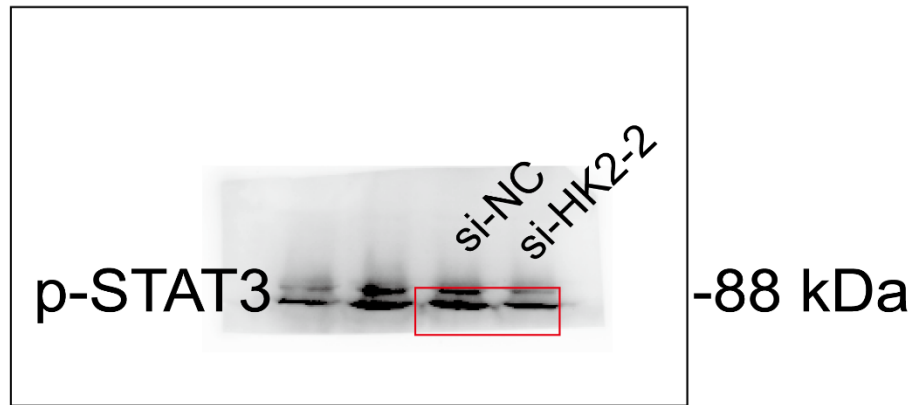

GBM1

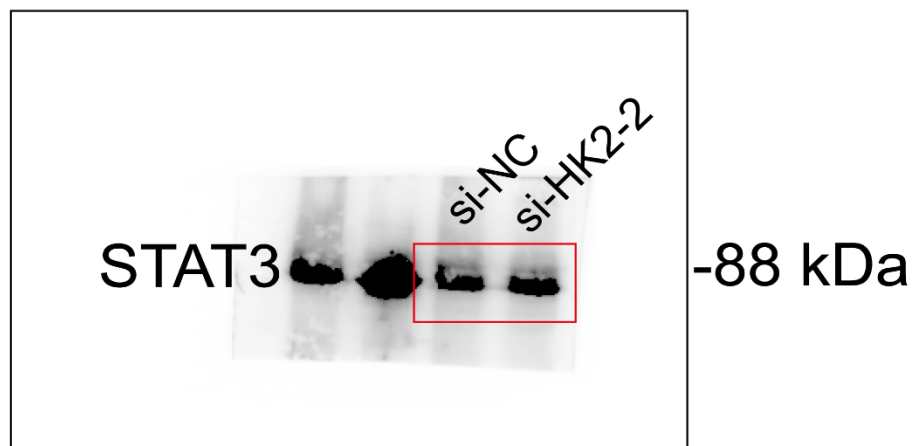

Figure 6

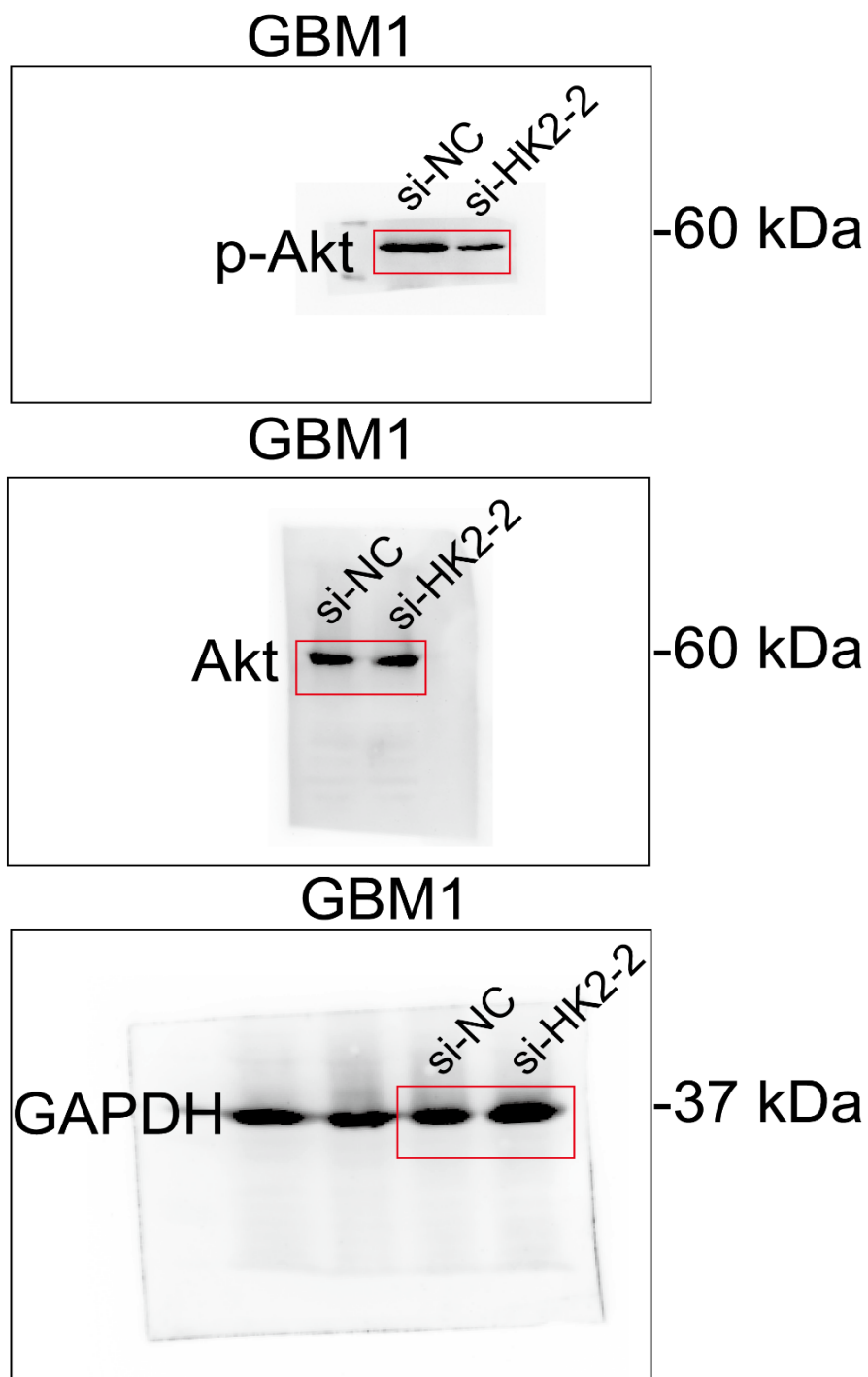

Supplement: Supplementary file 7 — Additional file 7:. The uncropped immunoblotting images of full-length blots. [file 12885_2022_10001_MOESM7_ESM.pdf]
