# Supplementary material for: The expression of Hexokinase 2 and its hub genes are correlated with the prognosis in glioma
Source: BMC Cancer. 2022 Aug 18;22:900. doi: 10.1186/s12885-022-10001-y (PMC9386956; doi:10.1186/s12885-022-10001-y)
Supplement: Supplementary file 8 — Additional file 8: Table S1. List of primer sequences for qRT-PCR. [file 12885_2022_10001_MOESM8_ESM.docx]

**Supplementary Table S1.** List of primer sequences for qRT-PCR.

| **Primer Names** | **Sequences (5′ to 3′)** |
| --- | --- |
| TNF-α | F：GAGTGACAAGCCTGTAGCCCATGTTGTAGC |
|  | R：GCAATGATCCCAAAGTAGACCTGCCCAGACT |
| IL-2 | F：ATGTACAGGATGCAACTCCTGTCTT |
|  | R：GTCAGTGTTGAGATGATGCTTTGAC |
| IL-3 | F：TCCAAACATGAGCCGCCTGCC |
|  | R：CATCAGAATGTCTTGGTCTTC |
| IL-4 | F：CCTCTGTTCTTCCTGCTAGCA |
|  | R：GCCGTTTCAGGAATCGGATCA |
| IL-12 | F：AGTGTCAAAAGCAGCAGAGG |
|  | R：AACGCAGAATGTCAGGGAG |
| *β*-Actin | F：CACTCTTCCAGCCTTCCTTCC |
|  | R：CGGACTCGTCATACTCCTGCTT |
