# Supplementary material for: The expression of Hexokinase 2 and its hub genes are correlated with the prognosis in glioma
Source: BMC Cancer. 2022 Aug 18;22:900. doi: 10.1186/s12885-022-10001-y (PMC9386956; doi:10.1186/s12885-022-10001-y)
Supplement: Supplementary file 9 — Additional file 9: Table S2. Different datasets to analyze HK2 expression in pathological classification of gliomas (ONCOMINE). [file 12885_2022_10001_MOESM9_ESM.docx]

**Supplementary Table S2.** Different datasets to analyze HK2 expression in pathological classification of gliomas (ONCOMINE).

| **Glioma Types** | **Fold Changes** | ***P*-value** | ***t*-test** | **Datasets** |
| --- | --- | --- | --- | --- |
| Glioblastoma (n = 542) | 5.631 | 1.85E-8 | 14.506 | TCGA Brain |
| Glioblastoma (n = 27) | 3.455 | 8.11E-5 | 6.873 | Bredel Brain 2 |
| Glioblastoma (n = 80) | 2.820 | 1.41E-4 | 7.815 | Murat Brain |
| Oligodendroglioma (n = 50) | 2.460 | 1.50E-6 | 5.268 | Sun Brain |
| Anaplastic Astrocytoma (n = 19) | 2.491 | 8.43E-5 | 4.200 | Sun Brain |
| Anaplastic Oligodendroglioma (n = 23) | 1.118 | 0.009 | 2.685 | French Brain |
