# Supplementary material for: The expression of Hexokinase 2 and its hub genes are correlated with the prognosis in glioma
Source: BMC Cancer. 2022 Aug 18;22:900. doi: 10.1186/s12885-022-10001-y (PMC9386956; doi:10.1186/s12885-022-10001-y)
Supplement: Supplementary file 10 — Additional file 10: Table S3. The information of clinicopathological characteristics of patient with gliomas. [file 12885_2022_10001_MOESM10_ESM.docx]

**Supplementary Table S3.** The information of clinicopathological characteristics of patient with gliomas.

| **Characteristics** | **Number of case (%)** |
| --- | --- |
| **Age (year)** |  |
| ≥40 | 224 (33.5) |
| <40 | 385 (57.5) |
| NA | 60 (9) |
| **Gender** |  |
| Male | 355 (53.0) |
| Female | 254 (38.0) |
| NA | 60 (9.0) |
| **Grades** |  |
| II | 226 (33.8) |
| III | 244 (36.5) |
| IV | 150 (22.4) |
| NA | 49 (7.3) |
| **IDH mutation status** |  |
| Yes | 429 (64.1) |
| No | 233 (34.8) |
| NA | 7 (1.10) |
| **MGMT promoter status** |  |
| Yes | 477 (71.3) |
| No | 161 (24.1) |
| NA | 31 (4.6) |
| **Transcriptome subtypes** |  |
| CL | 86 (12.9) |
| ME | 96 (14.3) |
| NE | 111 (16.6) |
| PN | 238 (35.6) |
| NA | 138 (20.6) |
| **Histology** |  |
| Astrocytoma | 194 (29.0) |
| Oligoastrocytoma | 130 (19.4) |
| Oligodendroglioma | 191 (28.6) |
| GBM | 152 (22.7) |
| NA | 2 (0.30) |
| **Vital states (at follow-up)** |  |
| alive | 428 (64.0) |
| Dead | 239 (35.7) |
| NA | NA (0.30) |
| **Chr.1p/19q co-deletion** |  |
| codel | 169 (25.3) |
| Non-codel | 494 (73.8) |
| NA | 6 (0.90) |
| **Expression of HK2** |  |
| Low expression | 335 (50.1) |
| High expression | 334 (49.9) |

CL, Classical; ME, Mesenchymal; NE, Neural; PN, Proneural; NA, Not available.
