# Supplementary material for: The expression of Hexokinase 2 and its hub genes are correlated with the prognosis in glioma
Source: BMC Cancer. 2022 Aug 18;22:900. doi: 10.1186/s12885-022-10001-y (PMC9386956; doi:10.1186/s12885-022-10001-y)
Supplement: Supplementary file 11 — Additional file 11: Table S4. Correlation between HK2 expression and clinicopathologic characteristics of glioma patients. [file 12885_2022_10001_MOESM11_ESM.docx]

**Supplementary Table S4.** Correlation between HK2 expression and clinicopathologic characteristics of glioma patients.

| **Characteristics** | **HK2 low expression** | **HK2 high expression** | ***P*-value** |
| --- | --- | --- | --- |
| **Age(year)** |  | | |
| ≥40 | 189 | 196 | 0.829 |
| <40 | 112 | 112 |  |
| **Gender** |  | | |
| Male | 175 | 180 | 0.940 |
| Female | 126 | 128 |  |
| **Grades** |  | | |
| II | 153 | 73 | <0.001 |
| III | 127 | 117 |  |
| IV | 31 | 119 |  |
| **IDH mutation status** |  | | |
| Yes | 274 | 155 | <0.001 |
| No | 60 | 173 |  |
| **MGMT promoter status** |  | | |
| Yes | 281 | 196 | <0.001 |
| No | 46 | 115 |  |
| **Transcriptome subtype** |  |  |  |
| CL | 30 | 56 | <0.001 |
| ME | 10 | 86 |  |
| NE | 80 | 31 |  |
| PN | 154 | 84 |  |
| **Histology** |  | | |
| Astrocytoma | 77 | 117 | <0.001 |
| Oligoastrocytoma | 78 | 52 |  |
| Oligodendroglioma | 149 | 42 |  |
| GBM | 30 | 122 |  |
| **Chr.1p/19q co-deletion** |  | | |
| codel | 152 | 17 | <0.001 |
| Non-codel | 183 | 311 |  |

CL, Classical; ME, Mesenchymal; NE, Neural; PN, Proneural.
