# Supplementary material for: The expression of Hexokinase 2 and its hub genes are correlated with the prognosis in glioma
Source: BMC Cancer. 2022 Aug 18;22:900. doi: 10.1186/s12885-022-10001-y (PMC9386956; doi:10.1186/s12885-022-10001-y)
Supplement: Supplementary file 12 — Additional file 12: Table S5. Univariate and multivariate analyses of various prognostics parameters in patients with glioma Cox-regression analysis. [file 12885_2022_10001_MOESM12_ESM.docx]

**Supplementary Table S5.** Univariate and multivariate analyses of various prognostics parameters in patients with glioma Cox-regression analysis.

|  | **Univariate analyses** | | | **Multivariate analyses** | | | |
| --- | --- | --- | --- | --- | --- | --- | --- |
|  | ***P*-value** | **Hazard Ratio** | **95% confidence interval** | | ***P*-value** | **Hazard Ratio** | **95% confidence interval** |
| **HK2** | < 0.001 | 1.791 | (1.573-2.039) | | 0.177 | 1.122 | (0.949-1.326) |
| **Grade** | < 0.001 | 4.732 | (3.821-5.837) | | < 0.001 | 2.498 | (1.828-3.412) |
| **IDH mutation status** | < 0.001 | 9.995 | (7.478-13.359) | | < 0.001 | 3.557 | (1.927-6.564) |
| **MGMT promoter status** | < 0.001 | 3.301 | (2.492-4.373) | | 0.919 | 1.020 | (0.701-1.483) |
| **Transcriptome subtype** | < 0.001 | 0.490 | (0.436-0.551) | | 0.435 | 0.929 | (0.772-1.118) |
| **Chr.1p/19q co-deletion** | < 0.001 | 4.598 | (2.906-7.276) | | 0.637 | 1.160 | (0.627-2.148) |
