# Supplementary material for: The expression of Hexokinase 2 and its hub genes are correlated with the prognosis in glioma
Source: BMC Cancer. 2022 Aug 18;22:900. doi: 10.1186/s12885-022-10001-y (PMC9386956; doi:10.1186/s12885-022-10001-y)
Supplement: Supplementary file 13 — Additional file 13: Table S6. The identification of the top 50 positively and negatively related genes with HK2. [file 12885_2022_10001_MOESM13_ESM.docx]

**Supplementary Table S6.** The identification of the top 50 positively and negatively related genes with HK2.

| The top 50 positively related genes | *HK2, SLC16A3, ALOX5AP, ALOX5, SLC1A5, SLC2A5, C3, LAPTM5, ITGB2, ELF4, SIGLEC9, LAIR1, SLC11A1, NAGA, LHFPL2, SERPINA1, CDCP1, RBM47, SYK, LAT2, HAVCR2, IL13RA1, SPI1, TLR2, NCKAP1L, ITGAM, CD53, FPR1, SASH3, TGFB1, FCGR2A, CD300A, CD86, GLIS3, COL8A2, RUNX1, RPS6KA1, SLC24A6, RAP1A, ARHGAP15, TNC, PIK3R5, TLR1, SLC7A7, NCF1, SAMSN1, FCER1G, CMTM7, GNAI3, ADORA3c.* |
| --- | --- |
| The top 50 negatively related genes | *MARCH11, USP11, CHGB, STAR, UNC80, CACNG2, LRRC20, RUNDC3A, FAM19A2, GDAP1, SCAMP5, SPIN2B, ALOX12B, NCRNA00219, LOC145837, TACC2, MADD, INSM2, HIP1R, GPR162, KIAA1409, SERP2, LRP4, TOMM20, KCNK3, SVOP, FAM123C, SPRN, CAMLG, GIT1, FXR2, AP3B2, TRIM67, RUNDC3B, CHRNB2, LOC283267, ECSIT, TMEM151B, FAM190A, CDK5R1, SPIRE2, KBTBD6, ZDHHC22, HAR1A, MARCH4, LETMD1, KCNIP2, MORN4, NECAP1, TPTE2P1.* |
