# Supplementary material for: The expression of Hexokinase 2 and its hub genes are correlated with the prognosis in glioma
Source: BMC Cancer. 2022 Aug 18;22:900. doi: 10.1186/s12885-022-10001-y (PMC9386956; doi:10.1186/s12885-022-10001-y)
Supplement: Supplementary file 14 — Additional file 14: Table S7. KEGG pathway enrichment analysis of HK2 related genes. [file 12885_2022_10001_MOESM14_ESM.docx]

**Supplementary Table S7.** KEGG pathway enrichment analysis of HK2 related genes.

| **Gene Set** | **Description** | **ES** | **NES** | ***P*-vaue** |
| --- | --- | --- | --- | --- |
| **hsa05150** | Staphylococcus aureus infection | 0.84893 | 2.1852 | 0 |
| **hsa05140** | Leishmaniasis | 0.79334 | 2.1417 | 0 |
| **hsa05134** | Legionellosis | 0.78192 | 2.0639 | 0 |
| **hsa04380** | Osteoclast differentiation | 0.72398 | 2.0476 | 0 |
| **hsa05133** | Pertussis | 0.75496 | 2.0438 | 0 |
| **hsa04610** | Complement and coagulation cascades | 0.75067 | 2.0304 | 0 |
| **hsa05145** | Toxoplasmosis | 0.71694 | 2.0253 | 0 |
| **hsa04640** | Hematopoietic cell lineage | 0.73545 | 2.0248 | 0 |
| **hsa04672** | Intestinal immune network for IgA production | 0.78363 | 2.0077 | 0 |
| **hsa05152** | Tuberculosis | 0.68548 | 1.9955 | 0 |
| **hsa04064** | NF-kappa B signaling pathway | 0.72037 | 1.9913 | 0 |
| **hsa04145** | Phagosome | 0.67911 | 1.9394 | 0 |
| **hsa04670** | Leukocyte transendothelial migration | 0.68956 | 1.9342 | 0 |
| **hsa05310** | Asthma | 0.79917 | 1.9151 | 0 |
| **hsa05169** | Epstein-Barr virus infection | 0.65655 | 1.9101 | 0 |
| **hsa05168** | Herpes simplex infection | 0.66098 | 1.9001 | 0 |
| **hsa04659** | Th17 cell differentiation | 0.67309 | 1.8732 | 0 |
| **hsa04611** | Platelet activation | 0.6656 | 1.8724 | 0 |
| **hsa04620** | Toll-like receptor signaling pathway | 0.67594 | 1.8674 | 0 |
| **hsa05144** | Malaria | 0.72403 | 1.8382 | 0 |
| **hsa04721** | Synaptic vesicle cycle | 0.61401 | -2.104 | 0 |
| **hsa05033** | Nicotine addiction | 0.76498 | 2.3622 | 0 |
| **hsa05130** | Pathogenic Escherichia coli infection | 0.69449 | 1.8153 | 0 |
| **hsa04621** | NOD-like receptor signaling pathway | 0.63137 | 1.8193 | 0 |
| **hsa04630** | JAK-STAT signaling pathway | 0.6286 | 1.8111 | 0 |
| **hsa04727** | GABAergic synapse | -0.4975 | 1.7516 | 0 |
| **hsa04723** | Retrograde endocannabinoid signaling | 0.46449 | 1.7601 | 0 |
| **hsa04724** | Glutamatergic synapse | -0.4772 | 1.7605 | 0 |
| **hsa04911** | Insulin secretion | 0.50152 | 1.7688 | 0 |
| **hsa04742** | Taste transduction | 0.48921 | 1.6749 | 0.0174 |
| **hsa04925** | Aldosterone synthesis and secretion | 0.47017 | -1.664 | 0 |
| **hsa04260** | Cardiac muscle contraction | 0.49747 | 1.6765 | 0 |

ES, enrichment score; NES, normalized enrichment score.
