# Supplementary material for: The expression of Hexokinase 2 and its hub genes are correlated with the prognosis in glioma
Source: BMC Cancer. 2022 Aug 18;22:900. doi: 10.1186/s12885-022-10001-y (PMC9386956; doi:10.1186/s12885-022-10001-y)
Supplement: Supplementary file 15 — Additional file 15: Table S8. The functional roles of HK2 positively related hub genes. [file 12885_2022_10001_MOESM15_ESM.docx]

**Supplementary Table S8.** The functional roles of HK2 positively related hub genes.

| **Genes** | **Gene description** | **Fold changes** | **Gene Summary** |
| --- | --- | --- | --- |
| ***ITGB2*** | integrin subunit beta 2 | 143 | Plays an important role in immune response |
| ***CD53*** | CD53 molecular | 95 | It contributes to the transduction of CD2-generated signals in T cells and natural killer cells. |
| ***C3AR1*** | complement C3a receptor 1 | 97 | Binding of C3a by the encoded receptor activates chemotaxis, granule enzyme release, superoxide anion production, and bacterial opsonization. |
| ***CYBB*** | cytochrome B-245 beta chain | 102 | It has been proposed as a primary component of the microbicidal oxidase system of phagocytes. |
| ***ITGAM*** | integrin subunit alpha M | 146 | The alpha M beta 2 integrin is important in the adherence of neutrophils and monocytes to stimulated endothelium. |
| ***CD33*** | CD33 molecule | 63 | Correlation with Acute Leukemia and Acute Promyelocytic Leukemia. |
| ***CLEC5A*** | C-type lectin domain containing 5A | 57 | Correlation with cell adhesion, cell-cell signalling, glycoprotein turnover, and roles in inflammation and immune response. |
| ***VAMP8*** | vesicle associated membrane protein 8 | 49 | Involved in the fusion of synaptic vesicles with the presynaptic membrane. |
| ***OLR1*** | oxidized low density lipoprotein receptor 1 | 44 | Involved in the regulation of Fas-and may play a role as a scavenger receptor. |
| ***CYBA*** | cytochrome B-245 alpha chain | 40 | Important for the microbicidal activity of these cells. |
