# Supplementary material for: The expression of Hexokinase 2 and its hub genes are correlated with the prognosis in glioma
Source: BMC Cancer. 2022 Aug 18;22:900. doi: 10.1186/s12885-022-10001-y (PMC9386956; doi:10.1186/s12885-022-10001-y)
Supplement: Supplementary file 16 — Additional file 16: Table S9. The functional roles of HK2 negatively related hub genes. [file 12885_2022_10001_MOESM16_ESM.docx]

**Supplementary Table S9.** The functional roles of HK2 negatively related hub genes.

| **Genes** | **Gene description** | **Fold changes** | **Gene Summary** |
| --- | --- | --- | --- |
| ***SYP*** | synaptophysin | 12 | This gene encodes an integral membrane protein of small synaptic vesicles in brain and endocrine cells. |
| ***CPLX1*** | complexin 1 | 7 | Among its related pathways are Neuroscience and Synaptic vesicle cycle. |
| ***SLC6A1*** | solute carrier family 6 member 1 | 10 | This gene include neurotransmitter: sodium symporter activity and gamma-aminobutyric acid: sodium symporter activity. |
| ***GABRG2*** | gamma-aminobutyric acid type A receptor subunit gamma2 | 9 | This gene includes chloride channel activity and GABA-A receptor activity |
| ***SCRT1*** | scratch family transcriptional repressor 1 | 7 | The encoded protein may promote neural differention and may be involved in cancers with neuroendocrine feature. |
| ***SV2A*** | synaptic vesicle glycoprotein 2A | 6 | The encoded protein may interact with synaptotagmin to enhance low frequency neurotransmission. |
| ***HTR1A*** | 5-hydroxytryptamine receptor 1A | 6 | This gene encodes a G protein-coupled receptor for 5-hydroxytryptamine (serotonin). |
| ***ACTL6B*** | actin like 6B | 6 | The protein is involved in diverse cellular processes, including vesicular transport, spindle orientation, nuclear migration and chromatin remodeling. |
| ***TMEM151B*** | transmembrane protein 151B | 5 | TMEM151B (Transmembrane Protein 151B) is a Protein Coding gene. |
| ***GABBR1*** | gamma-aminobutyric Acid type B receptor subunit 1 | 5 | This protein is the main inhibitory neurotransmitter in the mammalian central nervous system. |
